# Decellularised spinal cord matrix manipulates glial niche into repairing phase via serglycin‐mediated signalling pathway

**DOI:** 10.1111/cpr.13429

**Published:** 2023-02-18

**Authors:** Sheng Zhang, Man Zhai, Yiwei Xu, Jiandong Han, Jiaxin Chen, Yucui Xiong, Shihua Pan, Qizheng Wang, Chunlai Yu, Zilong Rao, Qi Sun, Yufei Sui, Ke Fan, Heying Li, Wenjing Guo, Cuicui Liu, Ying Bai, Jing Zhou, Daping Quan, Xiao Zhang

**Affiliations:** ^1^ Guangdong Engineering Technology Research Centre for Functional Biomaterials, School of Materials Science and Engineering Sun Yat‐sen University Guangzhou China; ^2^ CAS Key Laboratory of Regenerative Biology Guangzhou Institutes of Biomedicine and Health, Chinese Academy of Sciences Guangzhou China; ^3^ GMU‐GIBH Joint School of Life Sciences Guangzhou Medical University Guangzhou China; ^4^ School of Life Science and Technology University of Electronic Science and Technology of China Chengdu China

## Abstract

Astrocytes are the most abundant and widespread glial cells in the central nervous system. The heterogeneity of astrocytes plays an essential role in spinal cord injury (SCI) repair. Decellularised spinal cord matrix (DSCM) is advantageous for repairing SCI, but little is known regarding the exact mechanisms and niche alterations. Here, we investigated the DSCM regulatory mechanism of glial niche in the neuro‐glial‐vascular unit using single‐cell RNA sequencing. Our single cell sequencing, molecular and biochemical experiments validated that DSCM facilitated the differentiation of neural progenitor cells through increasing the number of immature astrocytes. Upregulation of mesenchyme‐related genes, which maintained astrocyte immaturity, causing insensitivity to inflammatory stimuli. Subsequently, we identified serglycin (SRGN) as a functional component of DSCM, which involves inducing CD44–AKT signalling to trigger human spinal cord‐derived primary astrocytes (hspASCs) proliferation and upregulation of genes related to epithelial–mesenchymal transition, thus impeding astrocyte maturation. Finally, we verified that SRGN‐COLI and DSCM had similar functions in the human primary cell co‐culture system to mimic the glia niche. In conclusion, our work revealed that DSCM reverted astrocyte maturation and altered the glia niche into the repairing phase through the SRGN‐mediated signalling pathway.

## Graphical abstract

Astrocytes play an essential role in spinal cord injury repair. However, the glial niche alterations during the extracellular matrix‐mediated repairing process concerning the decellularised spinal cord matrix (DSCM) regulatory mechanisms have not been clarified. In this study, we demonstrated that DSCM increased the proportion of differentiated neural progenitor cell subtypes in the migrated heterogeneous cell population of a spinal cord organotypic model, and this increase was correlated with a rise in immature astrocyte subtypes through ligand–receptor interactions. In addition, as a defined functional component of DSCM, which caused upregulated mesenchymal‐related gene expression via the serglycin (SRGN)‐mediated CD44–AKT signalling pathway. Thus, this SRGN‐mediated pathway sustained human spinal cord‐derived primary astrocytes in an immature status, reducing cytokine receptors' expression with declined response to inflammatory stimuli. Finally, we verify that defined SRGN‐COLI and DSCM had similar functions in the human primary cell co‐culture system to regulate astrocyte and endothelial cell subtypes in altering the glial niche into the repairing phase.

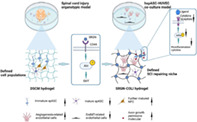

## 1 INTRODUCTION

As a repair mechanism in response to spinal cord injury (SCI), neural progenitor cells (NPCs) are activated and migrate toward damaged sites,^1^, ^2^ differentiate into multiple neural lineages, provide immunomodulatory factors and integrate into the remaining host neurons.^3^, ^4^ The SCI repair process can be accelerated using appropriate microenvironmental components to substitute the damaged extracellular matrix (ECM).^5^, ^6^, ^7^ However, the limited regenerative capability of endogenous nerve cells, and the complicity of cell types located around the lesion site limited the progress of repair efficacy.^8^ Hence, therapy must be optimized with the appropriate deployment of ECM components and cells to achieve further improved functional recovery during SCI repair. More research is needed to understand the multicellular level regarding the molecular mechanisms associated with ECM composition and function.

Astrocytes are the most abundant, widespread, and centralized glial cell types in the central nervous system (CNS).^9^ The astrocyte includes multiple subtypes with various functions that play beneficial or destructive effects during CNS injury repair.^10^, ^11^ After CNS injury is initiated, reactivating immature astrocyte properties promotes the endogenous repair processes, by generating a pro‐regenerative niche.^12^, ^13^, ^14^, ^15^ However, once reactive astrocytes are transformed into scar‐forming astrocytes and cause astrocytic scars, resulting in limited functional recovery as the chronic phase of SCI.^16^, ^17^ Astrocytes also coordinate crosstalk between neurons and endothelial cells (ECs), removing neurotransmitters to terminate synaptic transmission, re‐establish neuronal excitability, and preserve neuro‐glial‐vascular unit (NGVU) homeostasis.^18^

The ECM is involved in regulating NGVU homeostasis, triggering ECM–receptor interactions to activate downstream signalling pathways, including Src–FAK, PI3K–AKT, and Ras–MEK–MAPK. These pathways modulate multiple cell functions, such as focal adhesion dynamics, cellular proliferation, and differentiation.^19^, ^20^ Unsurprisingly, post‐injury changes to ECM functional composition alter ECM receptor‐mediated signalling. Research using a zebrafish model found that ECM activation of YAP signalling in post‐injury glial cells triggered epithelial‐to‐mesenchymal transition (EMT) and facilitated functional repair.^21^, ^22^, ^23^ Blocking the interaction between reactive astrocytes and Collagen I in a mouse spinal cord model, which inhibits astrocytic scar formation and promotes axon regeneration.^16^ Hence, studying the relationship between ECM changes and astrocyte cell fate helps clarify SCI's physical, biochemical, and biomechanical cues, and might provide new therapeutic strategies.

Previously, we demonstrated that utilizing decellularised spinal cord matrix (DSCM) as an injectable hydrogel promotes NPCs differentiation and facilitates cell–cell bridging in SCI.^5^ However, we understand very little regarding the specific functional composition, glial cell signalling activation, and glial niche alterations during DSCM‐driven SCI repair. We hypothesized that a component‐defined ECM shall alter multiple‐cell fate during SCI repair. Therefore, in this study, we performed single‐cell RNA sequencing (scRNA‐seq) for cellular subtypes identification, with biochemical assays to study how specific ECM components from DSCM manipulate glial niche mechanically using spinal cord organotypic and human glial‐vascular co‐culture model. We then fabricated serglycin (SRGN)‐COLI hydrogel as the defined substitutive ECM, and validated its function using a co‐culture model. The outcome shall contribute to a mechanistic understanding of SCI repair via ECM‐regulated glial niche from a molecular tissue biology point of view, and provides an ECM component‐defined approach for tissue repairing.

## 2 MATERIALS AND METHODS

### 2.1 Rat spinal cord organotypic isolation and culture

Spinal cord organotypic from SD (Sprague Dawley) rat (3 weeks old) was isolated and cultured on hydrogel refers to previously published work.^24^ Briefly, the rat was anaesthetised using avertin (2% wt/vol, i.p.) and dorsal laminectomy at T8–T10 was carefully performed. The T8–T10 spinal cord was isolated, and the animal was sacrificed by cervical dislocation. The isolated spinal cord was cut into thin slices (~1–2 mm), and then cultured on 0.5% wt/vol COLI /DSCM hydrogel with high glucose Dulbecco's Modified Eagle Medium (DMEM) with 10% Fetal bovine serum (FBS) and 1% Pen‐Strep.^24^

### 2.2 Preparation of COLI, DSCM, and SRGN‐COLI hydrogel (SRGN‐added COLI hydrogel)

High concentration of rat tail Collagen I (COLI) was used as control hydrogel (Corning no. 354249). The detailed coating procedure strictly followed the instruction manual. The final coating concentration is 5 mg/mL. COLI‐SRGN hydrogel was mixed with 5 mg/mL COLI hydrogel and 2.5 μg/mL SRGN recombinant human SRGN (Sino Biological, no. 13648‐H08H‐100). The detailed process and coating concentration (5 mg/mL) of DSCM hydrogel preparation is referred to our published literature.^5^

### 2.3 Human spinal cord astrocyte and human umbilical vein EC mono‐culture and co‐culture

Human spinal cord‐derived primary astrocytes (hspASCs) and human umbilical vein EC (HUVEC) were purchased from Sciencell (Human Astrocytes‐spinal cord: no. 1820, HUVECs; no. 8000). For monoculture, 200,000 cells were suspended with culture medium (Astrocyte medium, Sciencell, no. 1801; ECs medium: EGM2: Lonza no. CC‐3162) and were plated on the hydrogel‐coated 12‐well plate (100 μL hydrogel/well). For co‐culture, HUVECs and hspASCs co‐culture in a ratio of 3:1. For example, 200,000 cells (spASC, 150,000 cells; HUVEC, 50,000 cells) were suspended with EGM2 medium and were plated on the hydrogel‐coated 12‐well plate (100 μL hydrogel/well).

### 2.4 Cryo‐scanning electron microscope analysis

Cryo‐scanning electron microscope (SEM) analysis was performed as our previous protocol with minor modification.^25^ Microscope coverslips were coated with 5 mg/mL COLI, DSCM, or SRGN‐COLI hydrogel. After gelation, astrocyte cells were seeded on the hydrogel coated coverslip. The hydrogel materials or cells were washed with phosphate buffered saline (PBS) and ddH_2_O in sequence, then were fixed on the sample stage with conductive adhesive, and snap‐frozen with a liquid nitrogen slush bath. The sample stage was then placed in a cryo‐chamber (PP3010T, Quorum) and sublime for 30 min (Gel for 45 min, Gel+ cells for 30 min) at −80°C. After sputter coating with platinum (5 mA for 30 s), the hydrogel materials were transferred into the observation chamber and scanned with an SEM (GeminiSEM300, ZEISS) at −140°C.

### 2.5 Protein extraction and western blot

Total protein was extracted using RIPA buffer (Beyotime, P0013B). For Western blot, protein samples were separated by precast mini polyacrylamide gels (GenScript, M00657). After transferred to PVDF membranes, then blocked with 5% skim milk. Incubate the primary antibody overnight at 4°C. The detailed information of primary antibodies was listed in Table S1. The PVDF membranes were then washed with 0.1% TBST (three times for 10 min) and incubated with anti‐rabbit or anti‐mouse IgG, horseradish peroxidase (HRP) conjugate (1:10,000; Jackson ImmunoResearch, no. 111‐035‐003, no. 115‐035‐003) for 1 h at room temperature. The membranes were detected by the Western bright ECL kit (Advansta no. K‐12045‐D50) and quantified by ImageJ (v1.52a).

### 2.6 RNA extraction, reverse transcription, real‐time polymerase chain reaction, and bulk RNA sequencing

Total RNA was extracted by Trizol (Invitrogen, 15596018). Reverse transcription polymerase chain reaction (PCR) was carried out using GoScript™ Reverse Transcription Mix (Promega, A2790). quantitative real‐time PCR (qRT‐PCR) was performed using the SsoAdvanced Universal SYBR Green Supermix (Biorad, 1725275) on the step one system (The Applied Biosystems). Primers used in qRT‐PCR were listed in Table S2.

RNA‐Seq libraries were established, sequenced and *bioinformatic* analyses as described in our previous research.^25^

### 2.7 Immunofluorescence staining

Immunostaining and EdU assay was performed refer to our previously published work.^5^ Primary antibody information was listed in Table S3. Sample was washed with PBS for five times (5 min/time) and then incubated with secondary antibody (Table S3) for 1 h. After being washed with PBS, nuclear was stained with DAPI (1:5000). The image was captured by using Leica SP8 confocal microscope. Immunofluorescent signals and cell numbers were qualified using image J (v1.52a).

### 2.8 Hydrogel protein extraction and mass spectrometry analysis

The hydrogel was mixed with RIPA buffer (Beyotime, P0013B). The component of hydrogel was identified by mass spectrometry (MS) as described previously.^5^ The MS‐based proteomics data were normalized using the Normalyzer R package (version 1.12.0).^26^

### 2.9 Preparation of single‐cell suspension and single‐cell sequencing

Spinal cord slice culture system: After 12 days of cultivation, the organotypic was removed from the hydrogel, and hydrogel with cells was digested by collagenase (Sigma‐Aldrich, C9407) and trypsin (Thermo Fisher, 25200114). Digestion was neutralized with DMEM supplemented with 10% FBS. After centrifugation (300 g, 5 min), the cell pellet was washed and resuspension with PBS supplemented with 0.04% BSA. The cell numbers and viability (>75%) were confirmed using TC20 Automated Cell Counter (BioRad). In the hspASC‐HUVEC co‐culture system: After 5 days of cultivation, the approaches used for cell digestion and suspension preparation were identical to the Spinal cord slice culture system.

The single‐cell suspension was used to perform the scRNA‐seq using the 10× Genomics Chromium platform (Novogene). The detailed RNA extraction, library preparation, and sequencing procedure refer to our previously published paper.^25^

### 2.10 scRNA‐seq data pre‐processing and quality control

Droplet‐based scRNA‐seq raw reads were aligned and quantified by Cell Ranger software (version 5.0.1, 10× Genomics) against the GRCm38 and the GRCh38 reference genome respectively for the rat and human data. The output count data from Cell Ranger were then loaded into R (version 3.5.2) and processed using the R toolkit Seurat (version 3.2.3) for quality control, and cells were filtered based on parameters including the number of counts and features detected per barcode, the fraction of mitochondrial genes per barcode, the percentage of unique molecular identifiers (UMIs) mapping to ribosomal genes and genes typically known as housekeeping genes.

### 2.11 Differentially gene expression analysis, gene set variation analysis (GSVA), and cell‐type annotation

The Find All Markers function was performed to find differentially expressed genes between cells in a given cluster and all remaining cell, and we chose the “MAST” method, which is specifically tailored for scRNA‐seq data,^27^ for all the DE test. Furthermore, we performed the GSVA pathway analysis using the GSVA R package (version 1.34.0), and taking the Molecular Signatures Database (MSigDB)^28^ from the msigdbr R package (version 7.4.1) as the reference pathway panel. Combining the information from previously reported cell‐type‐specific marker genes, our top differentially expressed (DE) genes, and GSVA enrichment pathways, we assigned each cluster to a major cell type.

### 2.12 Diffusion map and pesudotime

The pesudotime trajectories were constructed using the diffusion pseudotime method^29^ by the destiny R package (version 3.0.1). The analysis was based on diffusion maps, which reduce dimensions by non‐linear transformations, so the non‐linear dynamics of underlyingly temporal processes are more likely to be captured by this method compared with that of principal component analysis (PCA).^30^

### 2.13 Cell–cell communication analysis

CellPhoneDB was used to investigate potential cell–cell communication regulated by ligand–receptor interactions from all cell types.^31^ Note that CellphoneDB was designed using human‐specific ligand–receptor interactions, so for the mouse data, we mapped human genes to their mouse orthologues before the CellPhoneDB analysis.

### 2.14 Proliferation scores and EMT scores analysis

The expression data of the common proliferation marker genes, identified by Whitfield et al.,^32^ was picked out from the normalized count matrixes, then the mean of the proliferation gene expression levels was taken as the proliferation score for a cell.

We scored EMT for all cells based on the epithelial and mesenchymal signatures and the EMT scoring method reported by a previous study.^33^

### 2.15 Akt inhibition and CD44 receptor blocking assay

The inhibition of Akt signalling was achieved with LY294002 (20 μM [1:500], 0.2% Dimethyl Sulfoxide (DMSO) used as a control) for 48 h. In addition, transfection of AKT1 small interfering RNA (sense: GCUAUUGUGAAGGAGGGUUTT, anti‐sense: AACCCUCCUUCACAAUAGCTT, 50 nM) and non‐targeting siRNA (50 nM) into astrocyte cell was performed by riboFECT CP Transfection Kit (Ribobio, C10511‐05). A total of 200,000 cells suspended in 250 μL of astrocyte medium were incubated for 30 min with 2 μg anti‐CD44 (mouse monoclonal, Proteintech, 60224‐1‐IG‐100UL), mouse IgG as a control (mouse IgG control, Genscript, A01007). Subsequently, cells were seeded on DSCM/COLI‐coated 12‐well plate. Following 48 h of culture, cells were subjected to subsequent analyses.

### 2.16 Statistical Analysis

All data from wet‐lab experiments were analysed by unpaired Student's *t*‐test using GraphPad Prism 8.0.1 statistical software (GraphPad Software). Data are shown as mean ± SEM.

## 3 RESULTS

### 3.1 DSCM enhances yield of mature NPC subtypes in the migrated heterogeneous cell population of a spinal cord organotypic model

**FIGURE 1:**
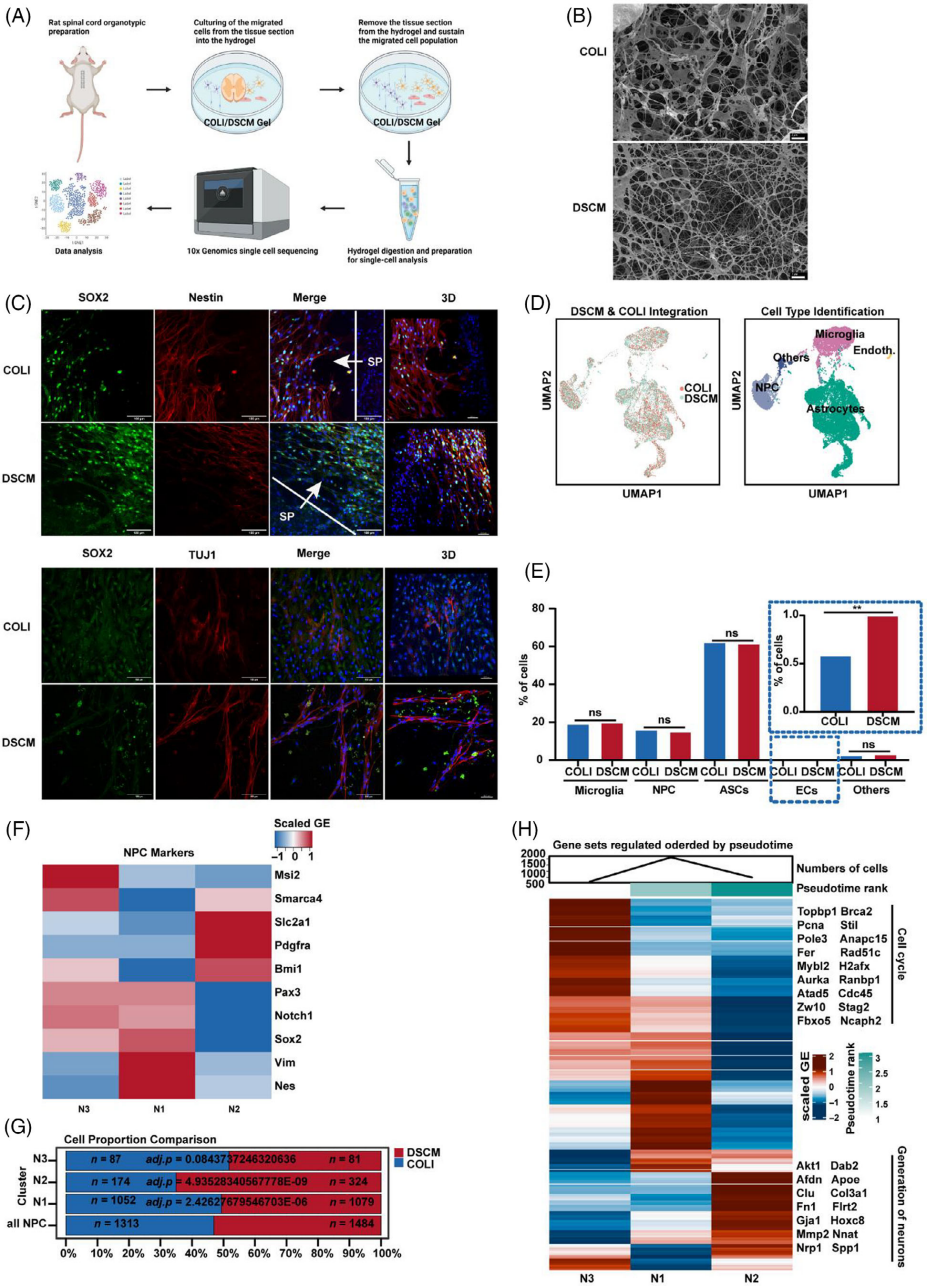
Single‐cell RNA sequencing (scRNA‐Seq) analysis of the migrated cell population from the rat spinal cord slice into decellularised spinal cord matrix (DSCM)/COLI cultured conditions reveals increased proportion of matured neural progenitor cells (NPCs) subtype. (A) The workflow shows the collection and processing of migrated cell from a rat spinal cord organotypic for scRNA‐seq (Created in BioRender.com). (B) Cryo‐SEM image of COLI and DSCM hydrogel (Bar = 3 μm). (C) Immunofluorescence staining (Nestin, SOX2, and TUJ1) of the cells that migrated from the spinal cord organotypic. (Scale Bar: 100 μm; Scale Bar: 50 μm for 3D; SP marks the position of spinal cord slice). (D) UMAP plot shows all cell types of migrated cells from a rat spinal cord organotypic under DSCM and COLI condition. (E) The relative proportion of each migrated cell type. **adjusted *p*‐value (*adj*. *p*) < 0.01; *ns* (no significance). The exact *adj*. *p* and cell number are shown in Figure S1E. (F) Heatmap shows the scaled gene expression of common NPC markers in each NPC sub‐populations. (G) Cell proportion of NPC subtypes. The exact *adj. p* and cell numbers (*n*) are shown on the graph (hypergeometric tests and Benjamini‐Hochberg correction). (H) Heatmap shows pseudotime rank and scaled gene expression of each NPC sub‐populations. The significantly enriched GO terms and gene names based on the top 50 high‐expression genes are pictured right. ASC, astrocytes; ECs, endothelial cells.

Previously, we found that DSCM injection enhanced endogenous cell migration to promote injury repair and mobility in rat SCI model.^5^ Here, we confirmed that DSCM treatment caused cell recruitment and migration to the injury site using tissue optical clearing technique and Paraffin‐embedded spinal cord sections for large‐volume spinal cord imaging (Figure S1A,H). To investigate the cues involved in DSCM‐driven SCI repair, we fabricated a rat spinal cord organotypic hydrogel in the bedding culture model (DSCM and COLI hydrogel; Figure 1A,B), to further understand the migrated cell population. After 12 days, cells had migrated from the tissue into the surrounding hydrogel and proliferated under both DSCM and COLI conditions (Figure S1B). The results of SOX2, Nestin, TUJ1, GLAST, and S100B staining on the migrated cells (Figures 1C and S1I,J) revealed that endogenous NPCs and astrocytes were present in the migrated population under both culturing conditions. Additionally, the DSCM condition had significantly longer axons (TUJ1^+^ cells) than the COLI condition (Figures 1C and S1C).

Subsequently, migrated cell types from the rat spinal cord organotypic model were identified using scRNA‐seq (Figure 1A). Utilization of uniform manifold approximation projection (UMAP) and PCA with reference markers (Microglia: *Ptprc* and *Vav1*, NPCs: *Sox2* and *Pax3*, Astrocyte: *Glul* and *Gja3*, ECs: *Pecam1* and *CD34*, Others: *Sox2*, *Pax3*, *Mag*, *Oligo1*) showed consistent grouping of five cell types in the dot and violin map (Figures 1D and S1D). Interestingly, apart from the EC group, cell population percentages did not differ (Figures 1E and S1E). We then proceeded to analyse cell‐type sub‐populations. Grouping analysis of NPCs yielded three subtypes: N1, N2, and N3 (Figure S1F); N1 and N3 expressed NPC reference markers (*Sox2*, *Pax3*, and *Nes*), whereas N2 expressed NPC reference markers (*Bmi1*, *Pdgfra*, and *Slc1a3*) (Figure 1F). The N1 and N3 subtypes were slightly less common in DSCM than in COLI, whereas N2 proportions were significantly higher in DSCM than in COLI (Figure 1G).

Next, we performed pseudotime trajectory analysis to understand lineage correlations in the three NPC subtypes. The results indicated that the N2 population was in a later lineage than the N3 population, with N1 in between (Figure 1H). Additionally, gene ontology (GO) enrichment analysis on the top 50 highly expressed genes showed that the N3 population expressed more genes related to the cell cycle and neuron generation (Figure 1H). These findings were consistent with GSVA clustering analysis, which revealed that the N2 population expressed genes related to neuron development and neurogenesis (Figure S1G). Overall, our analyses demonstrated that DSCM increased the proportion of more differentiated NPC cell subtypes than COLI.

### 3.2 DSCM increasing EGFR ^+^‐related immature astrocyte population to potentiate the communication with NPCs while the proportion of mature astrocyte decreased

**FIGURE 2:**
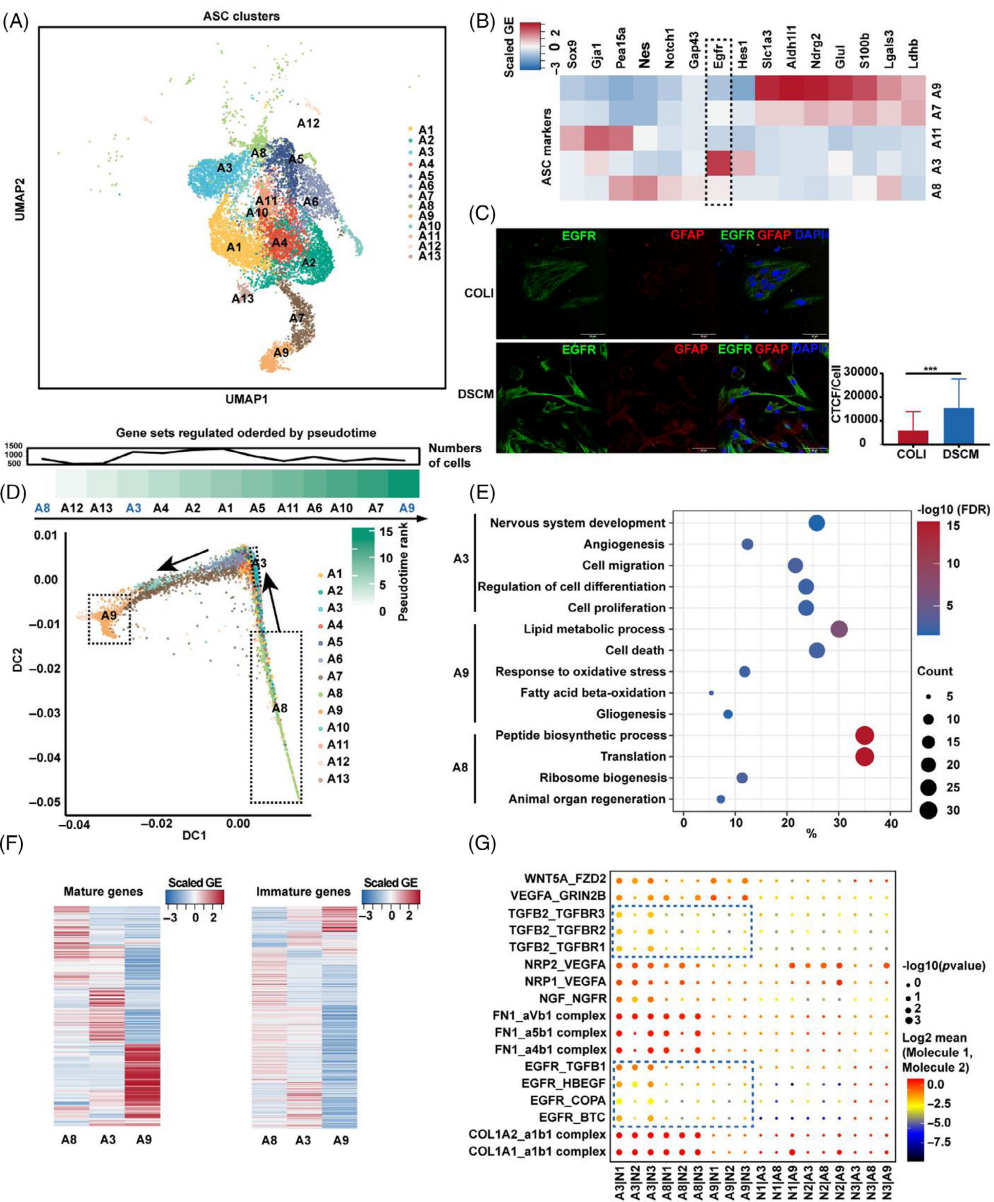
Single‐cell analysis revealed heterogeneity of migrated astrocytes (ASCs) population from rat spinal cord slice under decellularised spinal cord matrix (DSCM)/COLI conditions. (A) UMAP visualization of ASC sub‐clusters which migrated into DSCM/COLI hydrogel. (B) The scaled gene expression of reference ASC markers in ASC sub‐populations. (C) Immunofluorescence analysis and quantification of the migrated cell (EGFR and GFAP). Scale Bar: 50 μm. ***p < 0.001 (*t*‐test, mean ± SEM). (D) The pseudotime rank of each ASC subgroup. (E) Representative significantly enriched GO terms in ASCs subtype based on top 50 marker genes. (F) The scaled gene expression of reference ASC mature and immature gene sets. Representative ligand–receptor interactions between neural progenitor cell (N1, N2, and N3) and ASC subtypes (A3, A8, and A9) were shown in (G).

Astrocytes constituted the largest proportion of migrated cell types out of the rat spinal cord organotypic (Figure 1E). Scar‐formation astrocytes in the glial niche are believed to hinder nerve regeneration or express various factors that support axonal growth through EMT, but this function is controversial.^23^, ^34^ We thus characterized astrocytes in DSCM and COLI, respectively. Visualization in UMAP demonstrated that astrocytes were divided into 13 subtypes (Figure 2A). The DSCM culture had significantly more A3 and A8 subtypes than COLI culture, whereas COLI had higher percentages of A7, A9, and A11 (Figure S2A). Mature astrocyte makers (*Slc1a3*, *Glul*, and *S100b*) expressed in A7 and A9; in contrast, A3 and A8 expressed early astrocyte lineage markers such as *Egfr* and *Nes* (Figure 2B).^35^ We verified these findings in GFAP^+^ cells with the EGFR immunofluorescence staining was more evident in DSCM than COLI (Figure 2C).

Next, we performed pseudotime analysis over these 13 astrocyte subtype cells. The ranking of pseudotime indicated A8 as the earliest lineage within these 13 subtypes of ASCs, and A9 is the most mature form, relatively (Figure 2D). Included in the pseudotime rankings and cell proliferation fraction analysis, which showed that A8 and A3 had significantly higher proliferation scores than A9 (Figure S2B). Top 50 marker gene functional analysis indicated that A9 was associated with fatty acid metabolism, whereas A3 and A8 were associated with neuron‐related processes (Figure 2E). Additionally, mature or immature astrocyte reference marker gene expression in A9 was more relevant to the mature form; in contrast, A8 and A3 expressed more genes associated with the immature form (Figure 2F).^35^

Next, we used CellPhoneDB to analyse cell–cell communication based on the ligand‐receptor principle.^31^ At the intersecting pathways between astrocytes (A3, A8, and A9) and various NPCs, N1, N2, and N3 secreted different ligands to activate A3 via EGFR signalling (Figure 2G). In turn, the activated A3 secreted transforming growth factor beta 2(TGFβ2) to bind TGFBR of N1 and N3 (Figure 2G). This finding agrees with previous research showing that EGFR signalling in glial cells and TGFβ2 signalling pathway in stem cells respectively promote axonal regeneration and serotonergic neurons production.^36^, ^37^ Taken together, our results indicate that upregulation of the immature A3 population in DSCM culture is highly relevant to repair status. The A3 subtype may thus be a cue in SCI repair via ligand–receptor interactions with NPCs.

### 3.3 DSCM enhances immature gene expression of hspASCs and causes insensitive to the inflammatory stimulus through upregulating mesenchyme‐related genes

**FIGURE 3:**
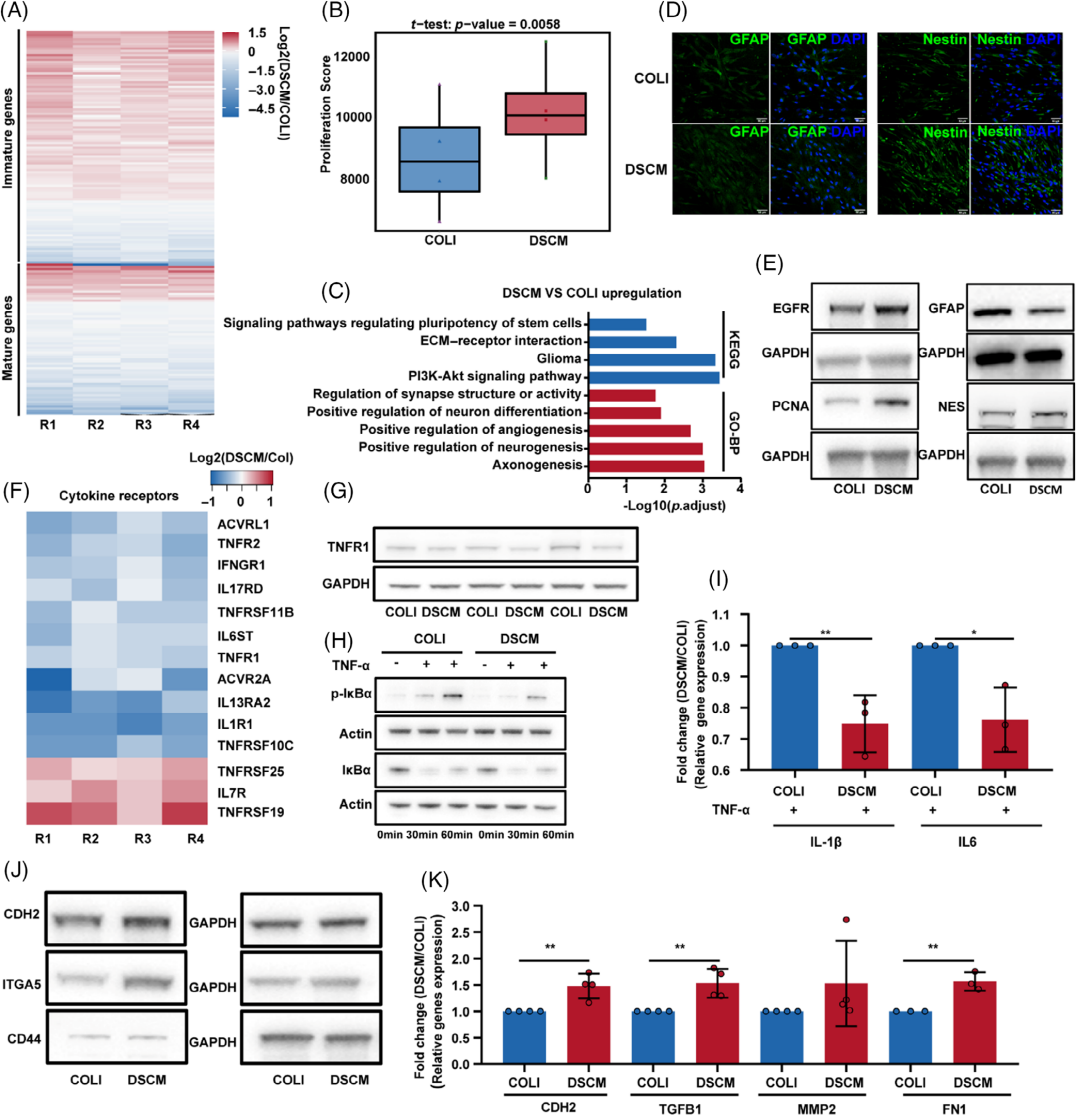
Decellularised spinal cord matrix (DSCM) promoted immature gene expression of human spinal cord‐derived primary astrocytes (hspASCs) and was insensitive to TNF‐α stimulation via increasing mesenchyme‐related genes expression. (A) Heatmap shows the significantly differentially expressed immature/mature‐related genes in hspASCs. The colour scale represents log2 (DSCM/COLI), and R1–R4 represent four biological repeats. (B) Proliferation score of hspASCs under DSCM/COLI condition. (C) Selective significantly up‐regulation enriched kyoto encyclopedia of genes and genomes (KEGG) pathway and GO terms in hspASCs cultured on DSCM. (D) Immunofluorescence analysis of GFAP and Nestin expression in hspASCs (Scale Bar: 50 μm). (E) Western blotting analysis of the protein expression in hspASCs (Immature marker: NES, EGFR; mature marker: GFAP; proliferation marker: PCNA). (F) Heatmap shows the significantly differentially expressed cytokine receptors in hspASCs. (G) Western blotting analysis of TNFR1 expression in hspASCs. (H) Western blotting analysis of p‐IκBα/IκBα level after 20 ng/mL TNF‐α stimulation in hspASCs. (I) Quantitative polymerase chain reaction (qPCR) measures the relative gene expression of *IL‐1β* and *IL6* in hspASCs after 24 h of 20 ng/mL TNF‐α stimulation. *p < 0.05; **p < 0.01 (*t*‐test, mean ± SEM). (J,K) Western blot and qPCR analysis of mesenchymal‐related gene expression in hspASCs. **p < 0.01 (*t*‐test, mean ± SEM). ECM, extracellular matrix.

Despite human and rat astrocytes having similar functions, the morphology of human astrocytes is more complex with unique transcriptional patterns,^38^ which caused the two species to respond differently to the same inflammatory stimuli.^39^ We thus performed bulk RNA‐seq assays on hspASCs and validated cell status in the associated niches, using mature or immature astrocyte reference gene sets in the literature to clarify DSCM regulatory mechanisms^35^: 75.8% of mature genes were downregulated and 72.4% of immature genes were upregulated in DSCM (Figures 3A and S3A). Additionally, the proliferation score analysis showed that hspASCs maintained a higher proliferative capacity when cultured in DSCM than when cultured in COLI (Figure 3B). Functional enrichment analysis of differentially expressed genes then showed that upregulated genes in DSCM‐cultured hspASCs were significantly correlated with the regulating pluripotency of stem cell signalling pathways. Enriched biological processes were involved in the positive regulation of angiogenesis, neurogenesis, and synapse structure or activity (Figure 3C). We then verified the bioinformatics findings using immunofluorescence, western blotting, and qPCR, demonstrated that Nes, Egfr, and PCNA were upregulated while GFAP down‐regulated in DSCM. (Figures 3D,E and S3B–E). These findings suggest that DSCM inhibited the mature form of hspASCs sustained immature status.

Astrocytes in geckos and foetal mice express the anti‐inflammatory protein Vav1,^40^ suggesting that immature mammalian astrocytes are insensitive to inflammatory stimuli. Therefore, we investigated whether DSCM prevents the hspASCs response to inflammation through increasing the proportion of immature hspASCs. First, we analysed expression profiles of inflammatory receptors in hspASCs under DSCM and COLI culture conditions. When compared with COLI, DSCM dramatically impaired the expression of inflammatory receptors, including classic TNFR1 (Figure 3F). Western blotting confirmed TNFR1 downregulation (Figure 3G and S3M). We then tested the hypothesis that differences in TNFR1 expression affect hspASC response to nuclear factor kappa B inhibitor alpha (TNF‐α) stimulation. Total IκB‐α concentration and degradation were comparable during the tumor necrosis factor alpha (TNF‐α) stimulation time course, indicating that DSCM impaired IκB‐α phosphorylation by ~2‐fold (Figures 3H and S3F,G). Further, qPCR showed that hspASCs response to TNF‐α stimulation under DSCM conditions had lower pro‐inflammatory cytokine interleukin*‐1β (IL‐1β)* and *interleukin‐6 (IL‐6*) expression (Figure 3I). In line with these results, RNA‐sequencing and functional enrichment analyses then indicated a downregulation of inflammation‐related signalling pathway in hspASCs response TNF‐α stimulation under DSCM condition (Figure S3H).

Another aspect of glial bridging formation during SCI repair is the activation of an EMT‐driven gene by immature astrocytes at the scar edge.^23^, ^34^ Thus, we investigated whether the repair characteristics of DSCM were mediated via spurring EMT regulation. Using published EMT gene sets as the reference marker, we compared DSCM and COLI cultures (Figure S3I).^23^ The analysis revealed that 83% of mesenchyme‐related genes were upregulated in hspASCs under DSCM conditions, whereas 62% of epithelial genes were upregulated under COLI conditions (Figure S3J). This result was cross‐validated with an EMT score assay using rat spinal cord organotypic scRNA‐seq data. Immature subtypes (A3 and A8) had significantly higher EMT scores than the mature subtype (A9; Figure S3K). Moreover, qPCR and western blotting confirmed that mesenchymal‐related marker genes were enhanced in hspASCs under DSCM, meaning EMT was enhanced in these culture conditions (Figures 3J,K and S3L). Taken together, these results indicate that DSCM culture altered hspASCs characteristics by inhibiting their maturation and increasing mesenchymal gene expression with sustained inflammatory insensitive profiling.

### 3.4 SRGN as a functional component of DSCM that sustains immature hspASCs status via activating the CD44–AKT signalling pathway

**FIGURE 4:**
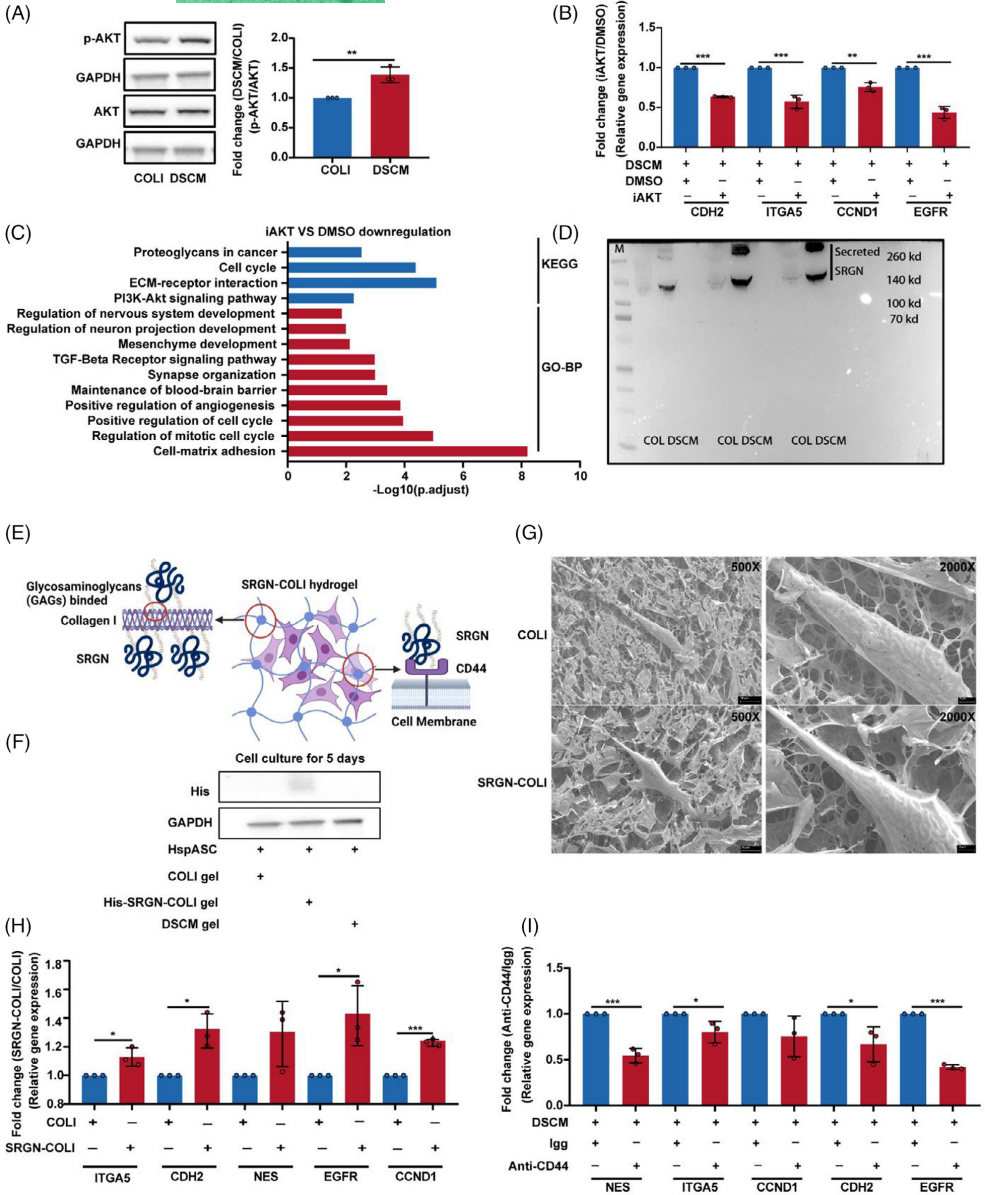
Decellularised spinal cord matrix (DSCM)‐regulated human spinal cord‐derived primary astrocytes (hspASCs) maturation via activation of serglycin (SRGN)–CD44–AKT signalling. (A) Western blotting and qualification show the level of p‐AKT in hspASCs under DSCM/COLI condition. **p < 0.01 (*t*‐test, mean ± SEM). (B) Quantitative polymerase chain reaction (qPCR) detects the relative gene expression of hspASCs incubated 48 h with LY294002 under DSCM condition. **p < 0.01; ***p < 0.001 (*t*‐test, mean ± SEM). (C) KEGG and GO enrichment analysis of significantly down‐regulation genes of hspASCs treated with LY294002 under DSCM condition. (D) Western blotting identifies the protein content of SRGN in DSCM/COLI hydrogel. The sample was obtained from three independent experiments. The total protein content loaded in each well is 40 μg. (E) Schematic of the principle of design SRGN‐COLI. (F) Western blotting analysis of the His‐SRGN level in hspASCs cultured on COLI, His‐SRGN‐COLI, and DSCM hydrogel for 5 days. (G) Cryo‐SEM image of hspASCs under COLI/SRGN‐COLI condition (Bar = 20 μm; Bar = 4 μm). (H) qPCR analysis of the relative gene expression in hspASCs under COLI/SRGN‐COLI condition (48 h). *p < 0.05; ***p < 0.001 (*t*‐test, mean ± SEM). (I) qPCR analysis of the relative gene expression of hspASC under DSCM condition with or without CD44 blocking. Mouse normal Igg was used as a control. *p < 0.05; **p < 0.01; ***p < 0.001 (*t*‐test, mean ± SEM).

In hspASCs, DSCM upregulated the expression of genes related to PI3K–AKT signalling compared with COLI (Figure 3C), but the exact mechanism was unclear. Here, we first verified that hspASCs cultured in DSCM had significantly higher AKT phosphorylation than hspASCs cultured in COLI (Figure 4A). We then added the AKT inhibitor LY294002 (iAKT) under DSCM culture conditions and determined the molecular characteristics of hspASCs using qPCR and RNA‐seq. The results of qPCR showed that iAKT treatment for 48 h significantly inhibited the expression of cell proliferation markers (*CCND1*), immature markers (*EGFR*), and mesenchymal markers (*CDH2* and *ITGA5*) (Figure 4B). Functional analysis of bulk RNA‐seq confirmed qPCR findings; downregulated genes were significantly enriched in the PI3K–AKT signalling pathway after iAKT treatment. Subsequently, we used AKT1 siRNA to verify this finding, which showed that AKT1 siRNA can significantly down‐regulate the expression of *AKT1*. Inhibiting *EGFR* and Glycolysis‐related rate‐limiting enzyme gene expression (Figure S4H), which is consistent with the previous study that the glycolysis pathway is impaired via inhibiting AKT signalling in hspASCs.^36^ In addition, GO analysis revealed that these genes were involved in regulation of nerve regeneration, angiogenesis, cell proliferation, and EMT (Figure 4C). In summary, the DSCM hydrogel culturing condition related to activation of AKT signalling, which increased the expression of mesenchymal and immature status‐related genes.

Previously, we investigated the overall composition of the core matrisome in the DSCM.^5^ Here, we aimed to determine the specific component in DSCM that potentiates AKT signalling in hspASCs. A comparison of peptide differences between DSCM and COLI hydrogels revealed distinct proteoglycans specifically (Figure S4A), which was confirmed with western blotting. Particularly, we identified proteins SRGN and Decorin (Figures 4D and S4B); notably, SRGN can interact with COLI through its glycosylation.^41^ This strong, non‐covalent interaction allows us to construct an SRGN‐GOLI hydrogel that should facilitate our mechanistic understanding of SRGN in the glial niche (Figures 4E). When we measured the elastic modulus of SRGN‐GOLI hydrogel, we found a difference of 117.4 Pa compared with COLI alone; which might cause insignificant alteration of cellular response (Figure S4D).^42^ Next, after 5 days of culturing hspASCs in SRGN‐COLI hydrogel (media changes every 48 h), SRGN levels were still detectable using an antibody against His‐SRGN (Figure 4F). The cellular morphology of hspASCs identified by Cryo‐SEM indicated that hspASCs exhibited stretched and embedded morphology regardless of SRGN presence or absence (Figure 4G).

Next, we used EdU staining to examine hspASCs proliferation in the SRGN‐COLI hydrogel. After 24 h of treatment, frames of arbitrarily selected images showed that EdU‐positive cells were significantly higher in the SRGN‐COLI hydrogel than in the COLI control conditions (Figure S4E). The SRGN‐COLI hydrogel also significantly increased AKT phosphorylation in hspASCs cultures (Figure S4C). We then used qPCR to investigate the effect of culturing hspASCs in SRGN‐COLI hydrogel for 5 days. We observed a significant increase in *CCND1*, *EGFR*, *CDH2*, and *ITGA5* expression compared with the COLI control (Figure 4H). Then, We used the SRGN‐targeted receptor CD44^43^ to examine the effects of a blocking antibody on ligand‐receptor interactions. Western blotting and qPCR results showed that blocking hspASCs with CD44 antibody significantly reduced AKT phosphorylation, along with CDH2, ITGA5, NES, and EGFR expression under both DSCM and SRGN‐COLI hydrogel culture conditions (Figures 4I and S4F,G). Taken together, these findings suggest that, mechanistically, SRGN is a functional component of DSCM, which potentiate cell proliferation and mesenchyme‐related gene expression through CD44 receptor activation via AKT signalling induction sustaining the immaturity of hspASCs.

### 3.5 The SRGN‐COLI hydrogel increased the proportion of hspASC subtypes that express axon growth‐permissive molecules in the hspASCs‐hECs co‐culture system

Angiogenesis and astrogenesis share similar stages during brain development, with more progenitor cells differentiating into both astrocytes and oligodendrocytes as blood vessels mature.^44^ Therefore, during SCI repair, ECs and communication between ASCs are essential. Hence, we investigated whether ECM alteration could regulate the fate of both ECs and ASCs in a simplified interaction model as a co‐culture system under SRGN‐COLI‐defined hydrogel compared with DSCMs in this human neurovascular unit model.

**FIGURE 5:**
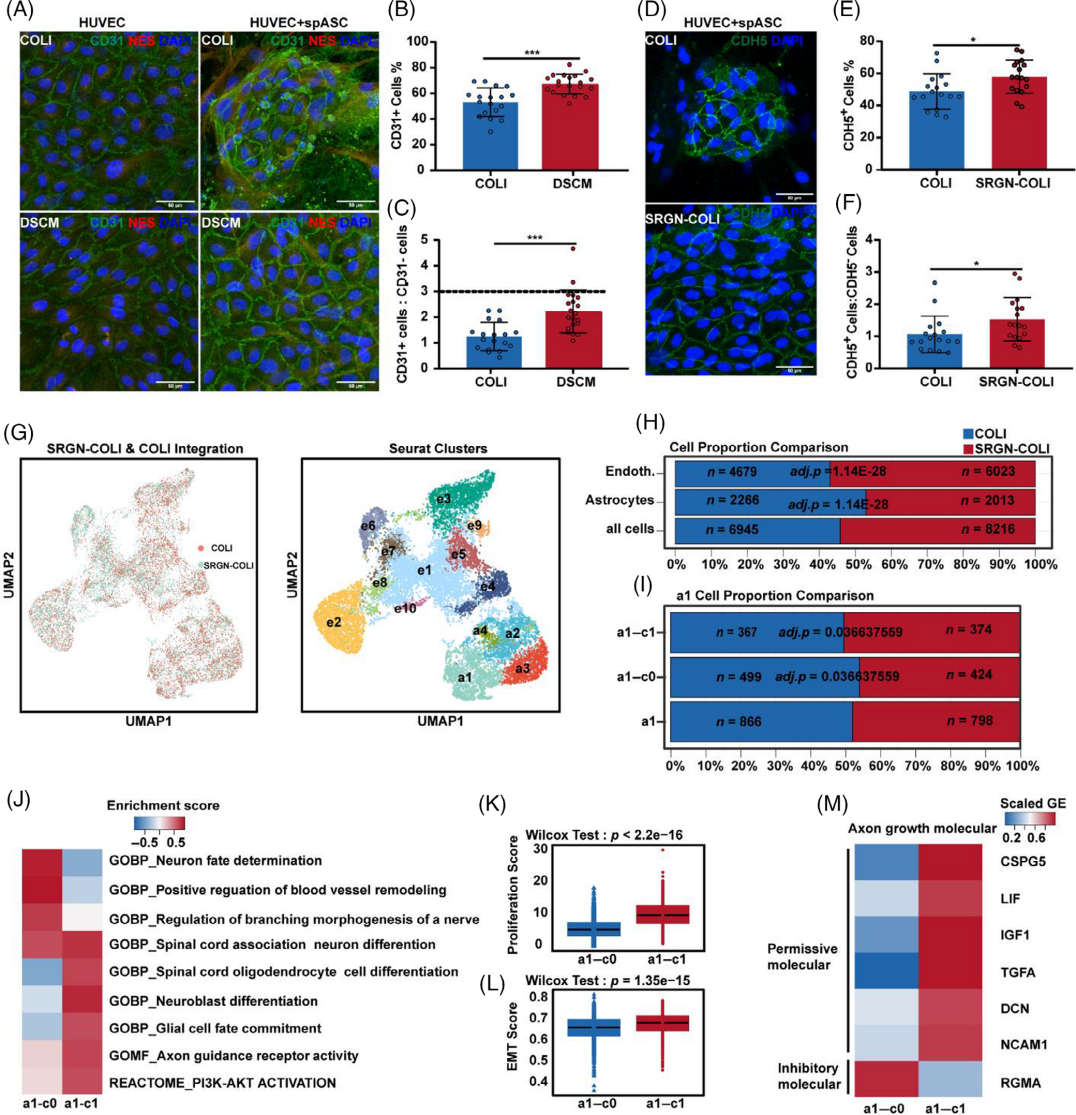
Characterization of hspASCs subtypes in hspASCs‐hECs co‐culturing model under different hydrogel conditions via single cell analysis. (A) Immunofluorescence analysis of the cell ratio in the hspASCs‐hECs coculture system. ECs marker: CD31; ASCs marker: Nes. Scale bar = 50 μm. (B,C) The quantification of CD31^+^/CD31^−^ cells. ***p < 0.001 (*t*‐test, mean ± SEM). (D) Immunostaining for CDH5 in the hspASCs‐hECs co‐culture model under COLI/SRGN‐COLI conditions. (E,F) The bar graph represents the proportion of CDH5^+^ cells in the hspASCs‐hECs co‐culture model. *p < 0.05 (*t*‐test, mean ± SEM). (G) UMAP plot integrates the scRNA‐seq data from hspASCs‐hECs co‐culture under COLI/SRGN‐COLI conditions. (H) The bar graph shows the cell proportion comparison of ECs and ASCs. Cell number (*n*) and adjusted *p*‐value were shown on the graph (hypergeometric tests and Benjamini–Hochberg correction). (I) The bar graph shows the cell proportion of the re‐cluster a1 subgroup. (Hypergeometric tests and Benjamini–Hochberg correction). (J) The heat map indicates representative significantly enriched GO terms and pathways of re‐cluster a1 subgroup by GSVA analysis. (K,L) Proliferation and EMT score of re‐clusters a1 subgroup (Wilcox test). (M) The significantly differentially expressed genes of axon growth inhibitory and permissive molecular in re‐cluster a1 subgroup. Fold changes of gene expression more than two‐fold were listed.

We first used qPCR to analyse the regulatory effect of DSCM on hECs (HUVECs), and found that DSCM conditions increased junction‐related gene (*TJP1*, *CLDN5*, and *PECAM1*) expression in HUVECs compared with COLI (Figure S5A). We then used hspASCs and HUVECs as in vitro human NGVU models for studying cell‐to‐cell interactions. The two cell types were seeded at a 1:3 ratio to form an endothelial sheet that simulates physiological conditions in the NGVU. In the DSCM co‐culture system, HUVECs formed a typical endothelial sheet at saturation confluence within 5 days (Figure 5A). In contrast, ECs formed clone‐like cell clusters in the COLI condition (Figure 5A), and the number of ECs co‐cultured in DSCM co‐culture resulted in significantly higher EC count than COLI culture (Figure 5B,C). To further verify that SRGN is a functional component in the hspASCs‐hECs co‐culture model, we co‐cultured hspASCs‐hECs for 5 days with COLI only or in the SRGN‐COLI hydrogel. Quantification of CDH5^+^ cells revealed similar numbers between the SRGN‐COLI hydrogel and DSCM culture, both of which were significantly higher than the value in COLI (Figure 5D–F).

We then applied scRNA‐seq to determine how cell fate changes in hspASCs‐hECs co‐culture versus the COLI and SRGN‐COLI conditions. An initial comparison of COLI and SRGN‐COLI using a UMAP plot combined with reference markers showed that co‐cultured cells formed different subclasses: hspASCs had four subtypes (a1–a4) and hECs had 10 subtypes (e1–e10; Figure 5G). The SRGN‐COLI condition resulted in significantly higher hECs proportions than COLI, whereas hspASCs proportion was significantly lower (Figure 5H). For the comparison between DSCM and COLI, the UMAP plot revealed that co‐cultured cells also formed two categories (Figure S5B,C). Moreover, hEC proportions were higher in DSCM than in COLI, while astrocyte proportions were lower (Figure S5D). Thus, SRGN‐COLI and DSCM have similar functions in regulating the hspASCs and hECs ratio, in contrast with COLI hydrogel alone.

To consolidate these findings, we analysed the regulatory effect of different hydrogels on astrocyte subtypes in the hspASCs‐hECs co‐culture system. We found that DSCM increased the proportion of co‐A1 subtypes (Figure S5E). These cells had higher proliferation and EMT scores than the other subtypes (Figure S5F,G), and expressed more immature astrocyte genes (Figure S5H). Furthermore, none of the remaining subtypes differed significantly in proportion between the SRGN‐COLI hydrogel and COLI conditions (Figure S5I). Differences in the gene clusters suggested that the immature a1 subtype can be categorized into additional subtypes: a1–c0 and a1–c1 groups. The proportion of a1–c1 cells was significantly higher in the SRGN‐COLI hydrogel condition than in COLI only, where a1–c0 cells were more abundant (Figure 5I). Further analysis using GSVA revealed that a1–c0 was mainly related to neural morphology and regulation of vascular remodelling, while a1–c1 was related to neural stem cell differentiation, glial cell fate determination, and axon guidance. Interestingly, a1–c1 also exhibited a stronger correlation with the PI3K–AKT activation signalling pathway (Figure 5J). Other characteristics included having higher proliferation and EMT scores than a1–c0 (Figure 5K,L), and expressed more axon growth permissive molecules (Figure 5M). These results indicate that the SRGN‐COLI hydrogel increased the proportion of cell subtypes that activated PI3K–AKT signalling. These subtypes have greater proliferative capacity and higher expression of axon growth‐permissive molecules.

### 3.6 The SRGN‐COLI hydrogel potentiates angiogenesis‐related EC subtypes and decreases EndoMT population in the hspASCs‐hECs co‐culture system

**FIGURE 6:**
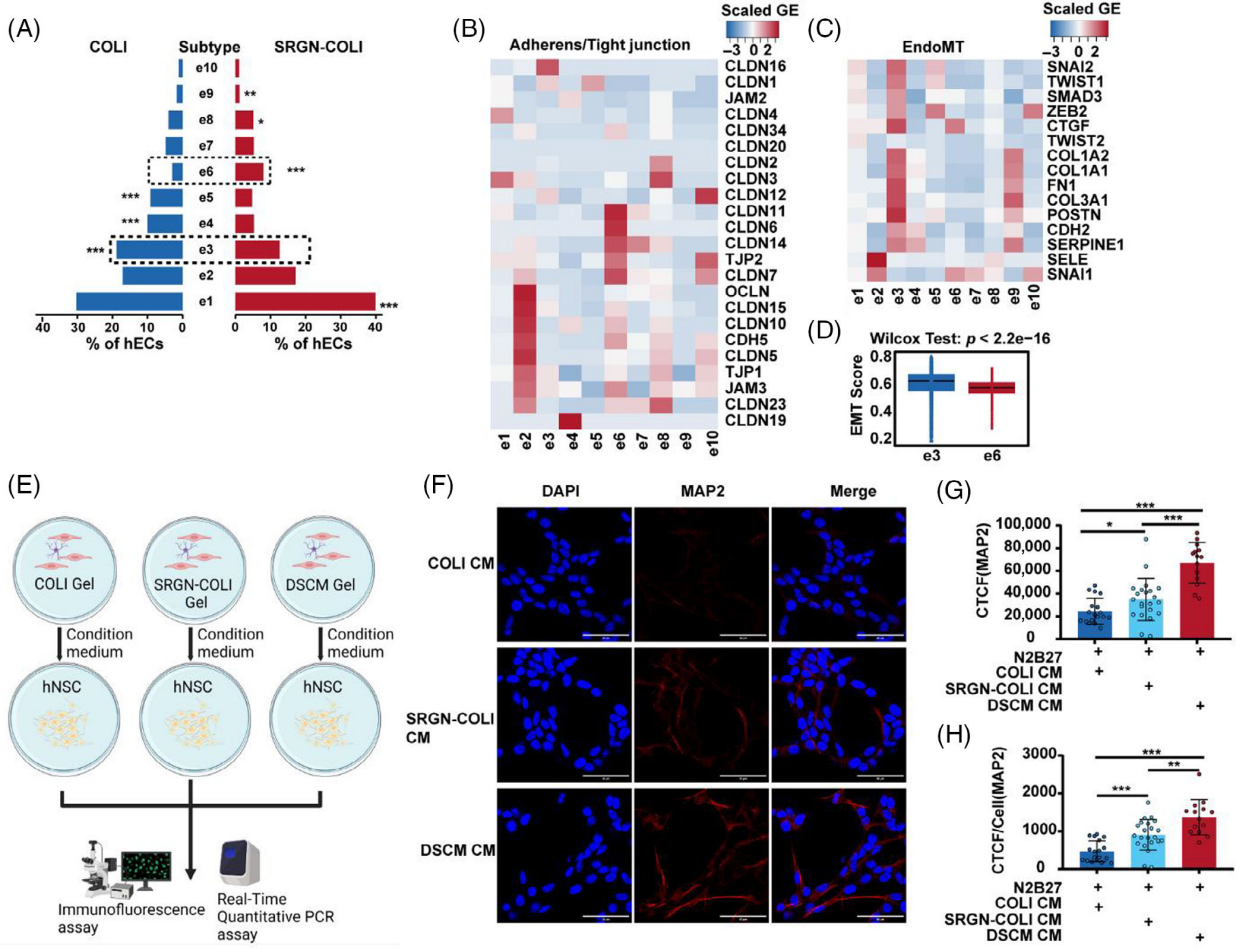
Characterization of serglycin (SRGN)‐COLI hydrogel altered the heterogeneity of hECs in the human spinal cord‐derived primary astrocyte (hspASC)‐human umbilical vein endothelial cell (HUVEC) co‐culturing model via single‐cell RNA sequencing analysis. (A) The proportion of ECs subgroups in hspASCs‐hECs co‐culture model under COLI/SRGN‐COLI condition. *adjusted *p* < 0.05; **adjusted *p* < 0.01; ***adjusted *p* < 0.001. The exact *p*‐value is listed in Figure S6E. (B,C) The heatmap shows the scaled gene expression level of adherens/tight junction genes and EndoMT marker genes in endothelial cells sub‐population. (D) Epithelial‐to‐mesenchymal transition score of e3 and e6 endothelial cell subgroup. (Wilcox test). (E) Diagram of the experimental design to test the conditional medium system for neural progenitor cells culturing. (F–H) Immunofluorescence analysis and qualification of MAP2 in hNSCs under different condition medium. Scale Bar: 50 μm. *p < 0.05; **p < 0.01; ***p < 0.001 (*t*‐test, mean ± SEM).

Vascular function is influenced by the phenotypic heterogeneity of vascular ECs in response to environmental stimuli.^45^ We therefore further examined the regulation of hECs fate under SRGN‐COLI culturing conditions, specifically testing e3 and e6 subtypes. We chose these two subtypes because scRNA‐seq showed that e6 had the greatest difference in cell ratio between SRGN‐COLI and COLI alone, whereas e1 and e3 had the highest proportions (Figure 6A). According to marker gene analysis, e6 highly expressed genes encoding EC‐adherent tight junction proteins, an essential component of angiogenesis and maintenance (Figure 6B). In contrast, scRNA‐seq showed that co‐E1 is more abundant in DSCM than in COLI, which is highly expressed in angiogenesis and Tip cell marker genes (Figure S6A,C), as consistent in GO analysis (top50) marker genes enrichment in angiogenesis and blood vessel related terms significantly (Figure S6B). Additionally, co‐E6 cultured in DSCM expressed more Endothelial to Mesenchymal Transition (EndoMT) genes (Figure S6C) and had a higher EMT score than the co‐E1 population (Figure S6D). Therefore, we also investigated EndoMT regulation by hECs cultured in SRGN‐COLI hydrogel. The results showed that e3—less abundant in SRGN‐COLI than in COLI—expressed more EndoMT genes and had a high EMT score (Figure 6C,D). We conclude that the SRGN‐COLI hydrogel is similar to DSCM hydrogel in promoting angiogenesis‐related EC subtypes and inhibiting EndoMT subtypes.

To understand the mechanisms underlying hEC subtypes variation in SRGN‐COLI hydrogel, we analysed communication between hspACSs and hECs using CellPhoneDB. The analysis yielded 835 interactions; in particular, four hEC subtypes (e1, e5, e7, and e10) interacted less with hspASCs than other EC subtypes (Figure S6E). Reflecting the results in Figure 5M, SRGN‐COLI enhanced axon growth‐permissive molecules. We thus hypothesized that the SRGN‐COLI hydrogel regulates cell fate through secretion factors. Next, we set up a conditional medium system to test the efficacy of COLI, SRGN‐COLI, and DSCM hydrogels as media for hspASCs‐hECs co‐culture. We added hspASCs‐hECs co‐culture medium to human primary neural stem cells (hNSCs) differentiation medium, then used immunofluorescence and qPCR to detect the expression of cell differentiation markers (Figure 6E). Immunofluorescence revealed that the medium derived from DSCM and SRGN‐COLI hydrogel significantly increased expression of the mature neuron marker MAP2 in hNPCs (Figures 6F–H and S7). The qPCR results showed that the DSCM hydrogel significantly increased *ASCL1*, *MAP2*, *TUJ1*, and *NeuroD1* expression. *ASCL1*, *MAP2*, and *TUJ1* also slightly increased in the SRGN‐COLI hydrogel, but with batch‐to‐batch variation (Figure S6F). In summary, both the SRGN‐COLI and DSCM hydrogels can regulate the NGVU niche and alter NPCs fate through influencing factors secreted by the cultured cells.

## 4 DISCUSSION

**FIGURE 7:**
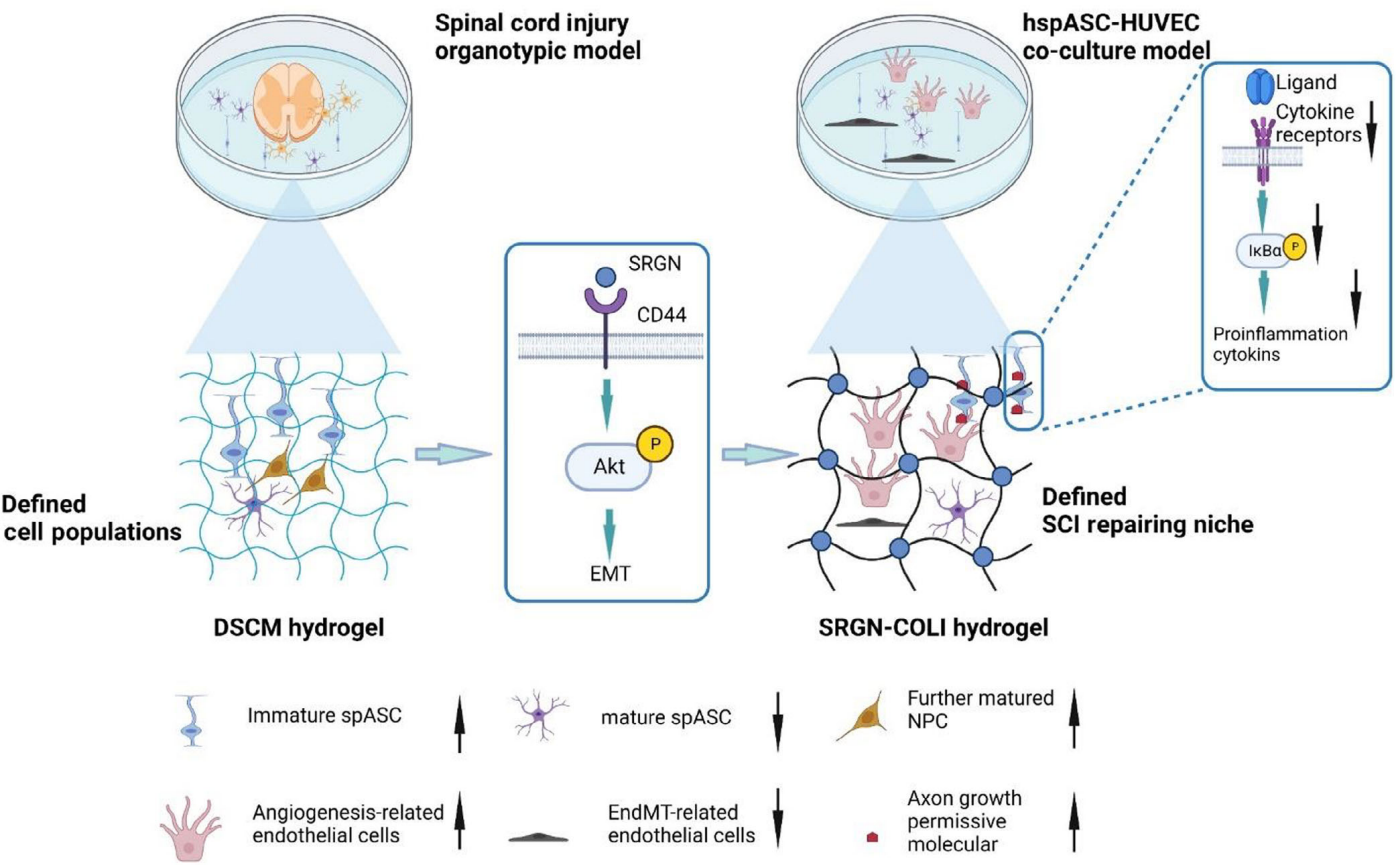
A model to show the proposed mechanism of decellularised spinal cord matrix (DSCM)‐manipulated glial cell niche during DSCM‐driven spinal cord injury (SCI) repair process and the defined SCI repairing niche construction. EMT, epithelial‐to‐mesenchymal transition; HUVEC, human umbilical vein endothelial cell; NPC, neural progenitor cell; SRGN, serglycin.

In this study, we investigated ECM regulatory mechanisms in the NGVU between DSCM and COLI. With our rat spinal cord organotypic model, we demonstrated that DSCM increased the proportion of differentiated NPC subtypes in the migrated cell population. This increase was linked with a rise in immature astrocyte subtypes through ligand–receptor interactions. The mechanism of DSCM's effects upregulating mesenchymal gene expression, thus maintaining hspASCs in an immature state that is insensitive to inflammatory stimuli. Our proteomics analysis and biochemical experiment then confirmed that SRGN is one of the functional components behind DSCM potentiation of cell proliferation and EMT alteration in hspASCs, with the specific pathway being AKT signalling to activate the CD44 receptor. The SRGN‐COL hydrogel was further evaluated using the hspASCs‐hECs co‐culture model and conditioned medium with human hNSCs. These findings verified that the DSCM and SRGN‐COLI culturing conditions have similar advantages in converting glial niche into a repairing phase. Both increased the proportion of angiogenesis‐related cell subtypes for endothelial tip cell sprouting, inhibited the EndoMT population, and facilitated differentiation of NPCs (Figure 7).

Previous research demonstrated that DSCM promotes NPC differentiation,^5^ but the cell fate changes in subtypes of glial niche were unknown. Our single‐cell analysis revealed enrichment of GO terms related to mature and differentiated NPC subtype N2 (Figures 1H and S1G), limited mature neuronal cells were identified using the reference marker (Figure 1C). As reported reactivating immature astrocyte enhance the regenerative potential of the CNS injury.^14^, ^15^, ^46^ Moreover, the reverting astrocyte maturation to an neural stem cell‐like state has the same role.^46^, ^47^ In our study, pesudotime results indicated that astrocyte subtype A8 slightly overlapped with the NPC N2 subtype at the start and endpoints of the two lineage branches (Figure S2C), suggesting that the two subtypes are derived from the same progenitors.

Moreover, studies using the zebrafish model showed that the subjects possess a type of immature glial cell that activates the EMT regulatory network to form the glial bridge.^23^ In contrast, astrocytes in the SCI mouse model were limited to the EMT‐driven gene regulatory network and did not promote glial bridge formation.^23^ Nevertheless, these cells have physiological functions similar to those of immature astrocytes and bridging glial cells in zebrafish. These prior findings agreed with our results from culturing hspASCs in DSCM (Figure 3J,K), as well as in rat spinal cord organotypic scRNA‐seq data (Figure S3K). Hence, we propose that DSCM provides an advantageous ECM environment through increasing the expression of mesenchymal gene sets, inhibiting hspASC maturation, and raising the proportion of immature hspASCs. In response, axons are regenerated, promoting glial niche repair in SCI.

The physical barrier formed by ECs is essential for maintaining NGVU homeostasis of via cell junction.^48^ Our data demonstrated that DSCM promotes upregulation of junction‐related genes in ECs (Figure S5A). Moreover, hspASCs‐hECs co‐culture in DSCM and SRGN‐COLI hydrogel increased the tight junction proportion of EC subtypes, but decreased the proportion of fibrosis‐related EndoMT cell subtypes (Figures 6B–D and S6C,D). Macrophages can inhibit EndoMT in ECs via downregulating TGFβ1.^49^ Here, our findings suggested that the decrease in EndoMT under DSCM conditions was because fewer mature astrocyte subtypes were present, and TGFβ1 release was inhibited (Figure S6G,H). The resultant ratio of different astrocyte subtypes preserves homeostasis of the NGVU niche.

Multiple studies have found a link between WNT5a and neural repair. The protein promotes glioma cell proliferation,^50^ the number of Ki67^+^ aberrant astrocytes increased significantly in an amyotrophic lateral sclerosis (ALS) rat model,^51^ and WNT5a protein expression was upregulated in the spinal cord of ALS patients,^52^ These results suggest that the increase in aberrant astrocytes in ALS may be related to the increased expression of WNT5a. Consistently, we found that the EndoMT‐related EC subtypes co‐E6/e3 secretes WNT5a and interacts with different astrocyte subtypes (Figure S6G,H). Thus, DSCM's inhibitory effects on hspASC proliferation may occur through inhibiting EndoMT EC subtypes, which then results in WNT5a downregulation in hspASC‐hEC co‐culture system.

Naturally derived decellularised ECM (dECM) has been used in tissue engineering and regenerative medicine for SCI repair.^5^, ^6^, ^7^ However, owing to the complexity of its composition, many unknowns remain regarding the regulatory mechanism of dECM during tissue repair. To address this issue, we used comparative proteomics and biochemical verification to identify SRGN as a functional component of DSCM (Figure 4D). SRGN acts through its receptor CD44, and we determined the role of CD44/AKT signalling in hspASCs under different culture conditions (Figure 4). Different cell types have distinct CD44 isoforms^53^ that can alter cell–cell interactions. For example, CD44 Splice Variant V8–V10 (CD44V8–V10) overexpression in the metastatic melanoma line Lu1205 induces junction disassembly and VE‐cadherin phosphorylation in ECs.^54^ Thus, the patterns we observed in our study are likely the result of separate pathway activation by CD44 isoforms across various cell types.

The mechanistic focused understanding of SRGN in a defined system caused the limitation of systematic SRGN investigation in vivo. The function of SRGN also affects other tissue‐specific cell types.^55^, ^56^, ^57^, ^58^ However, a systematic understanding of the effect on various cell types at the SCI site was understudied. On the other hand, the SRGN‐fabricated hydrogel acts as the ECM microenvironment, interacting with different types of cells in vivo. Therefore, as the complicated situation in vivo, it is not appropriate to use the COLI‐SRGN system to study the mechanism of SRGN over astrocytes in vivo. SRGN is one of the functional components that regulate the fate of astrocytes under DSCM culturing, and it did not emphasize that SRGN is the only functional component. In order to overcome this limitation, the construction of SRGN for astrocyte‐specific knockout in future might be required.

In conclusion, this study improved the current understanding of the mechanisms underlying changes to cell fate during DSCM‐driven SCI repair. These findings suggested that these mechanistic advantages of SRGN as a functional component of DSCM provided microenvironment are consistent with the SRGN‐COLI culturing condition for converting the glial niche into the repairing phase.

## AUTHOR CONTRIBUTIONS

*Conceptualization*: Sheng Zhang, Xiao Zhang, and Daping Quan. *Methodology*: Man Zhai, Chunlai Yu, Heying Li, Wenjing Guo, Cuicui Liu, and Ke Fan. *Investigation*: Sheng Zhang, Yiwei Xu, Jiandong Han, Jiaxin Chen, Shihua Pan, Yucui Xiong, Qizheng Wang, Zilong Rao, Yufei Sui, and Qi Sun. *Visualization*: Sheng Zhang, Xiao Zhang, Yiwei Xu, and Man Zhai. *Supervision*: Xiao Zhang, Daping Quan, Ying Bai, and Jing Zhou. *Writing—original draft*: Sheng Zhang and Xiao Zhang. *Writing—review and editing*: Sheng Zhang, Yiwei Xu, Daping Quan, and Xiao Zhang.

## FUNDING INFORMATION

This work was supported by the National Natural Science Foundation of China 52073314 (to Daping Quan), Scientific Instrumentation Development Program of Chinese Academy of Science ZDKYYQ20210006 (to Xiao Zhang) and ZDZBGCH2018005 (to Xiao Zhang), and Science and Technology Plan Foundation of Liaoning O0312001 (to Ke Fan).

## CONFLICT OF INTEREST STATEMENT

The authors declare that they have no conflicts of interest.

## Supporting information


**Data S1:** Supporting information

## Data Availability

All bulk and single cell transcriptomic data are available in the NCBI Gene Expression Omnibus (GSE202733). All data needed to evaluate the conclusions in the article are present in the article and/or the Supplementary Materials. All bioinformatic software used in this publication is open source.
