# Supplementary material for: Decellularised spinal cord matrix manipulates glial niche into repairing phase via serglycin‐mediated signalling pathway
Source: Cell Prolif. 2023 Feb 18;56(9):e13429. doi: 10.1111/cpr.13429 (PMC10472524; doi:10.1111/cpr.13429)
Supplement: Supplementary file 1 — Data S1: Supporting information [file CPR-56-e13429-s001.docx]

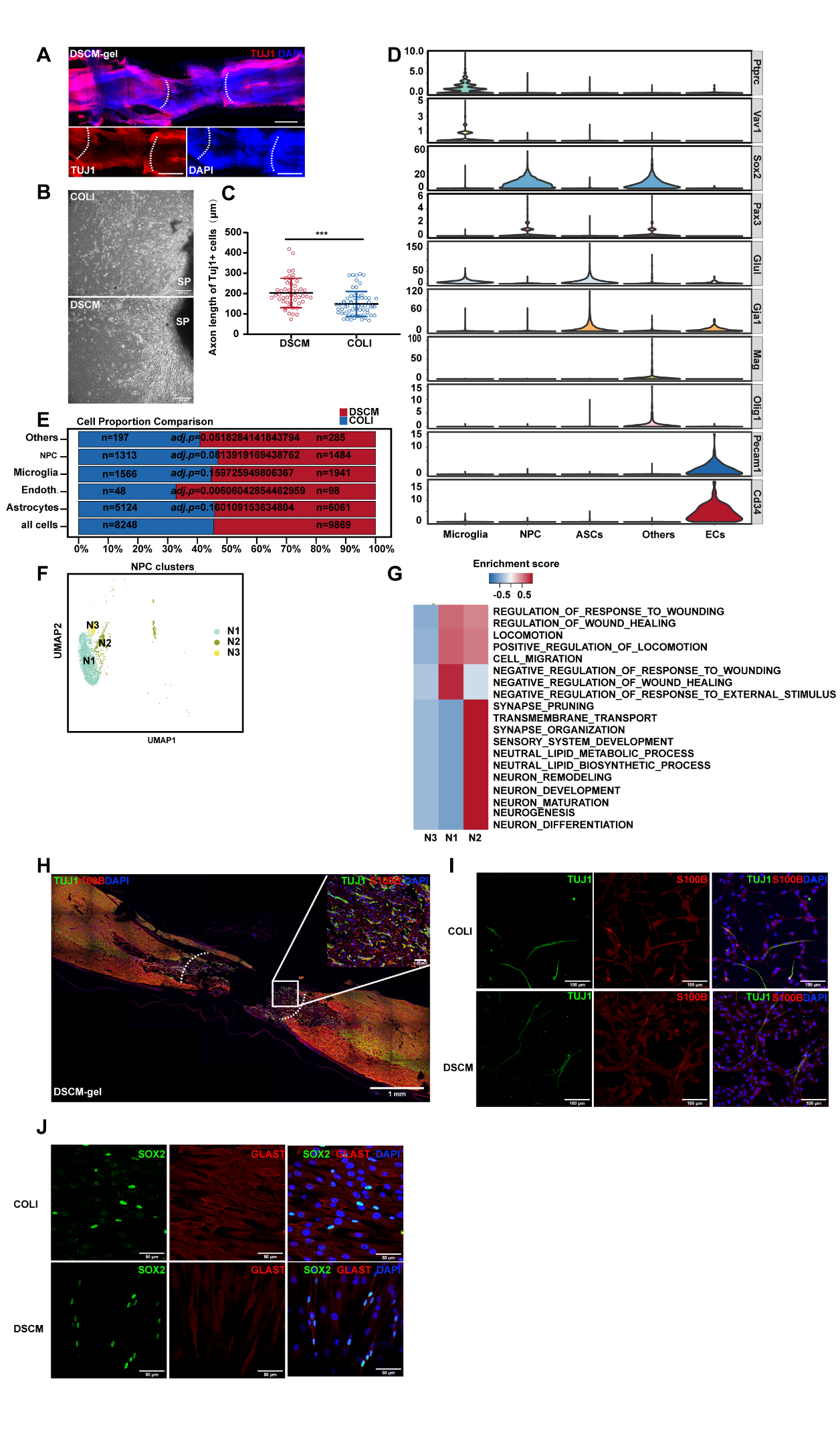


**Fig. S1. The heterogeneity of cell types of the cell migrated out of the spinal cord slice.** (**A**) Immunofluorescence staining (TUJ1) of the spinal cord from the DSCM treated spinal cord transection injury model (Bar= 1 mm). (**B**) Brightfield's picture shows the cells that migrated out from the spinal cord slice. (**C**) Axon length of TUJ1^+^ cells under DSCM/COLI condition, related to figure 1D. ***, *p*<0.001(*t*-test, mean ± SEM). (**D**) Violin plot shows the marker genes of each migrated cell type. (**E**) Comparative assessment of the proportion of each cell type migrated out of the spinal cord slices. The *adj. p* was calculated based on hypergeometric tests and corrected with Benjamini-Hochberg correction. (**F**) UMAP visualization of all NPC subtypes under DSCM/COLI conditions. (**G**) Neuron-related GO terms were identified using GSVA analysis in NPC subtypes. **(H)** Immunofluorescence staining (TUJ1 and S100B) of the spinal cord from the DSCM treated spinal cord contusion model. (I), (J) Immunofluorescence staining (TUJ1, S100B, SOX2 and GLAST) of the cell that migrated from the spinal cord organotypic.


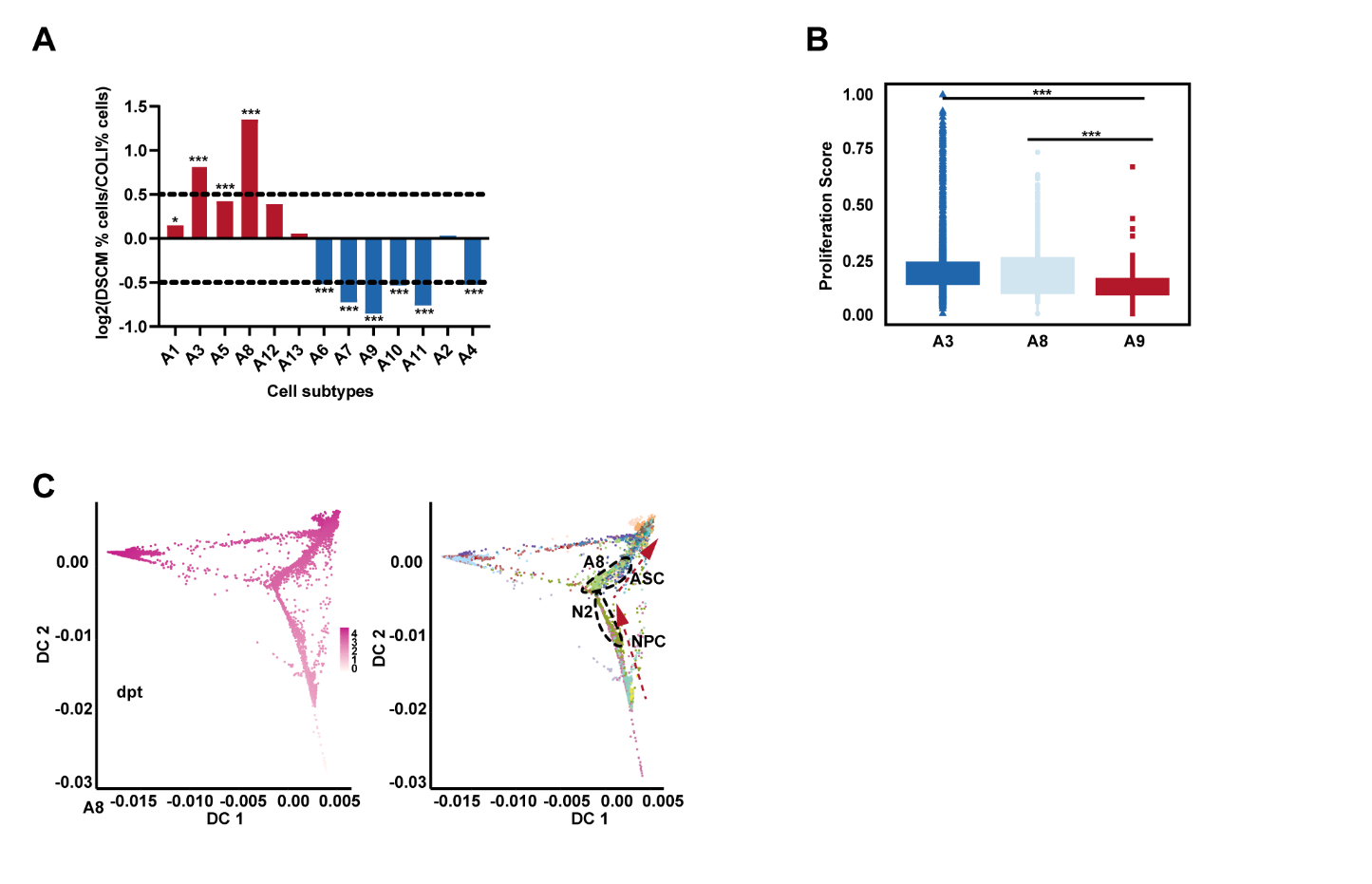


**Fig. S2.** **Characterization of ASCs subpopulation of the cell migrated into DSCM/COLI hydrogel. (A)** Comparative assessment of the proportion of each ASCs subtype under DSCM/COLI culturing. *, *adj. p* < 0.05; ***, *adj. p*<0.001 (hypergeometric tests and Benjamini-Hochberg correction). (**B**) Proliferation scores of A3, A8, and A9. ***, *p*<0.001 (Wilcox test). (**C**) Pseudotime analysis of all clusters migrated into DSCM/COLI hydrogel. Dotted boxes represent A3 and A8 spASCs subtypes, and the red arrow indicates pseudotime trajectory.


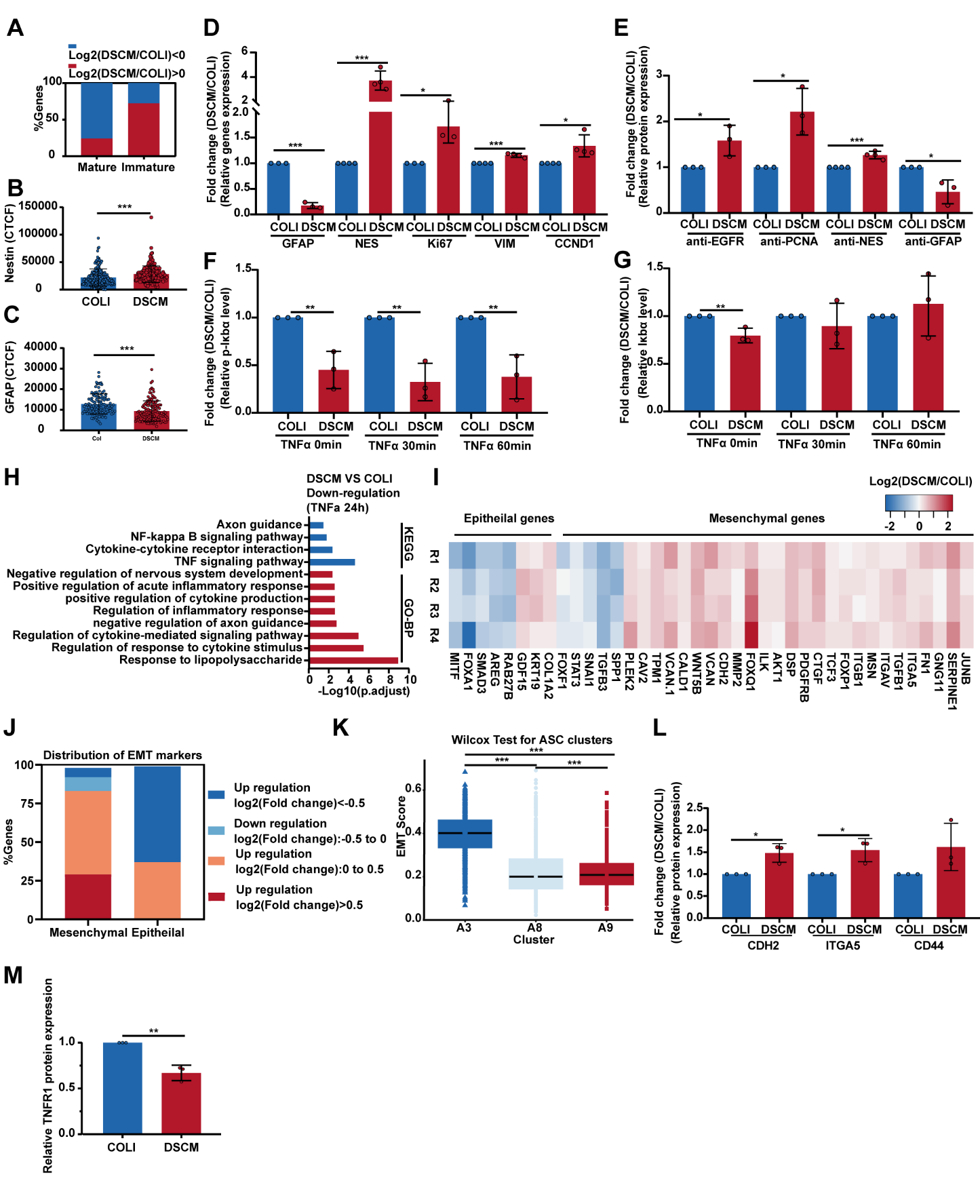


**Fig. S3. DSCM increases the immaturity subpopulation by enhancing the EMT gene expression in hspASCs.** (**A**) Distribution of immature and mature-related genes using bulk-seq. (**B**), (**C**) Quantification of GFAP and Nestin immunofluorescence staining in Fig. 3D. ***, *p*<0.001 (*t*-test, mean ± SEM). (**D**)Relative gene expression of immature marker (*VIM,* *NES*), mature marker (*GFAP*), and proliferation marker (*Ki67*, *CCND1*) in hspASCs. *, *p*<0.05; ***, *p*<0.001 (*t*-test, mean ± SEM) (**E**) Quantification of the western blotting related to figure 3E. *, *p*<0.05; ***, *p*<0.001 (*t*-test, mean ± SEM). (**F**), (**G**) Quantitative assessment of the western blotting in Fig. 3H. **, *p*<0.01 (*t*-test, mean ± SEM) (**H**) Particular significance enriched the KEGG pathway and GO term of the down-regulation genes in DSCM after 24 hours of 20 ng/mL TNF-α incubation. (**I**) Heatmap represents the relative expression of EMT-related genes. Scale indicated log2 (DSCM/COLI). (**J**) Distribution of mesenchymal and epithelial-related genes. (**K**) The EMT score of ASCs subpopulations in the rat spinal cord organotypic model. ***, *p*<0.001 (Wilcox test). (**L**) Quantification of the western blotting in figure 3J. (**M**) Quantification of the western blotting in Fig. 3G. **, *p*<0.01 (*t*-test, mean ± SEM).


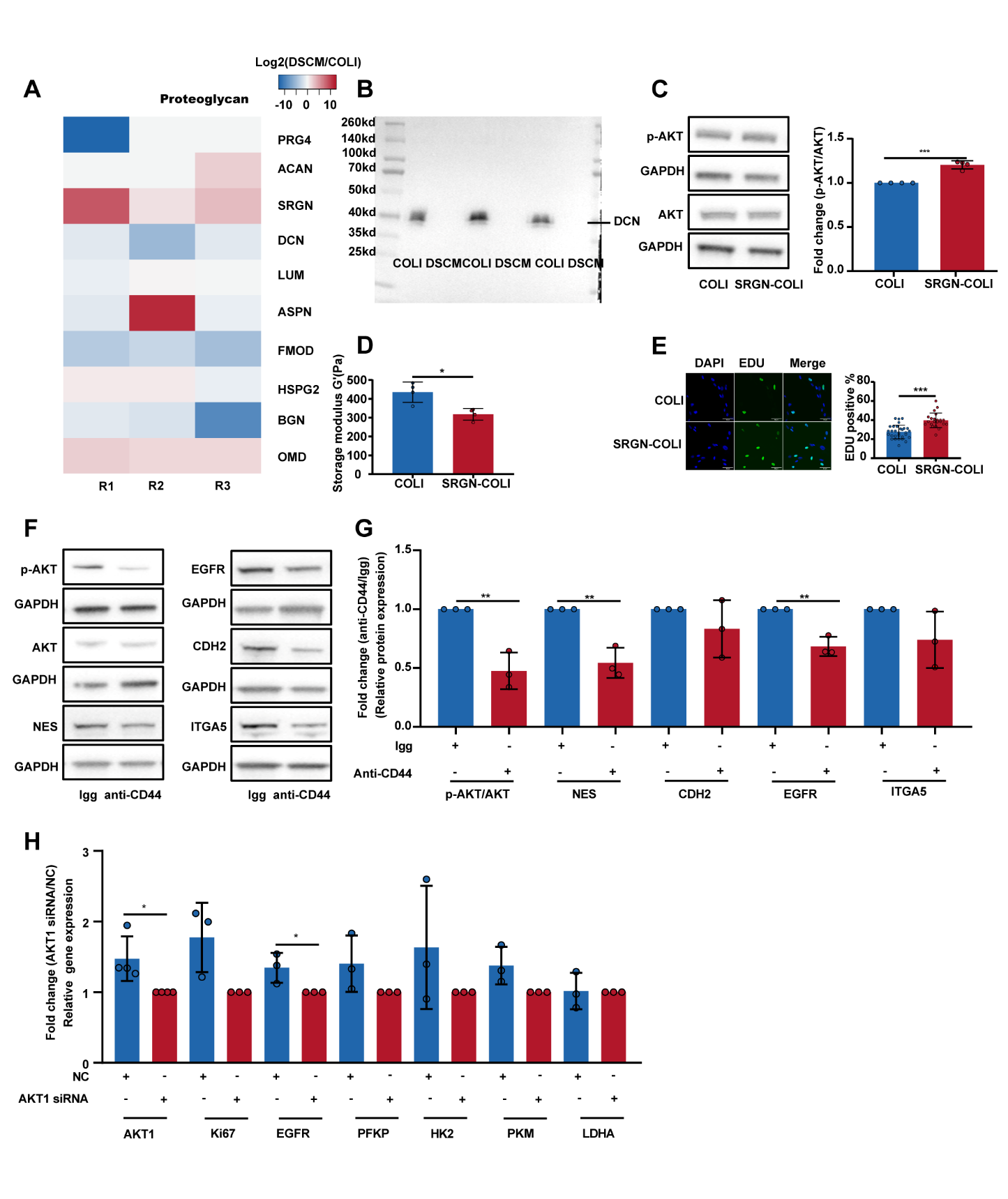


**Fig. S4. SRGN is one of the defined functional compositions of DSCM.** (**A**) Mass spectrum analysis identifies the content of proteoglycan in COLI/DSCM hydrogel. The colour scale represents Log2(DSCM/COLI). (**B**) The protein content of DCN was detected by Western blotting. 40μg total protein was loaded in each well. (**C**) Western blotting analysis of the phosphorylation level of AKT (p-AKT) in hspASCs under the COLI and SRGN-COLI condition. ***, *p*<0.001 (*t*-test mean ± SEM). (**D**) The storage modulus G′ of COLI and SRGN-COLI hydrogel. *, *p*<0.05 (*t*-test, mean ± SEM). (**E**) EdU fluorescence staining and quantification of hspASCs culture
under COLI/SRGN-COLI condition. ***, *p*<0.001 (*t*-test, mean ± SEM). (**F**) and (**G**) Western blotting analysis of the relative protein expression in CD44 blocked hspASC under the COLI/SRGN-COLI conditions. **, *p*<0.01 (*t*-test, mean± SEM). (**H**) qPCR detects the relative gene expression of hspASCs transfected with AKT1 siRNA under DSCM condition, *, *p*<0.05 (*t*-test, mean ± SEM).


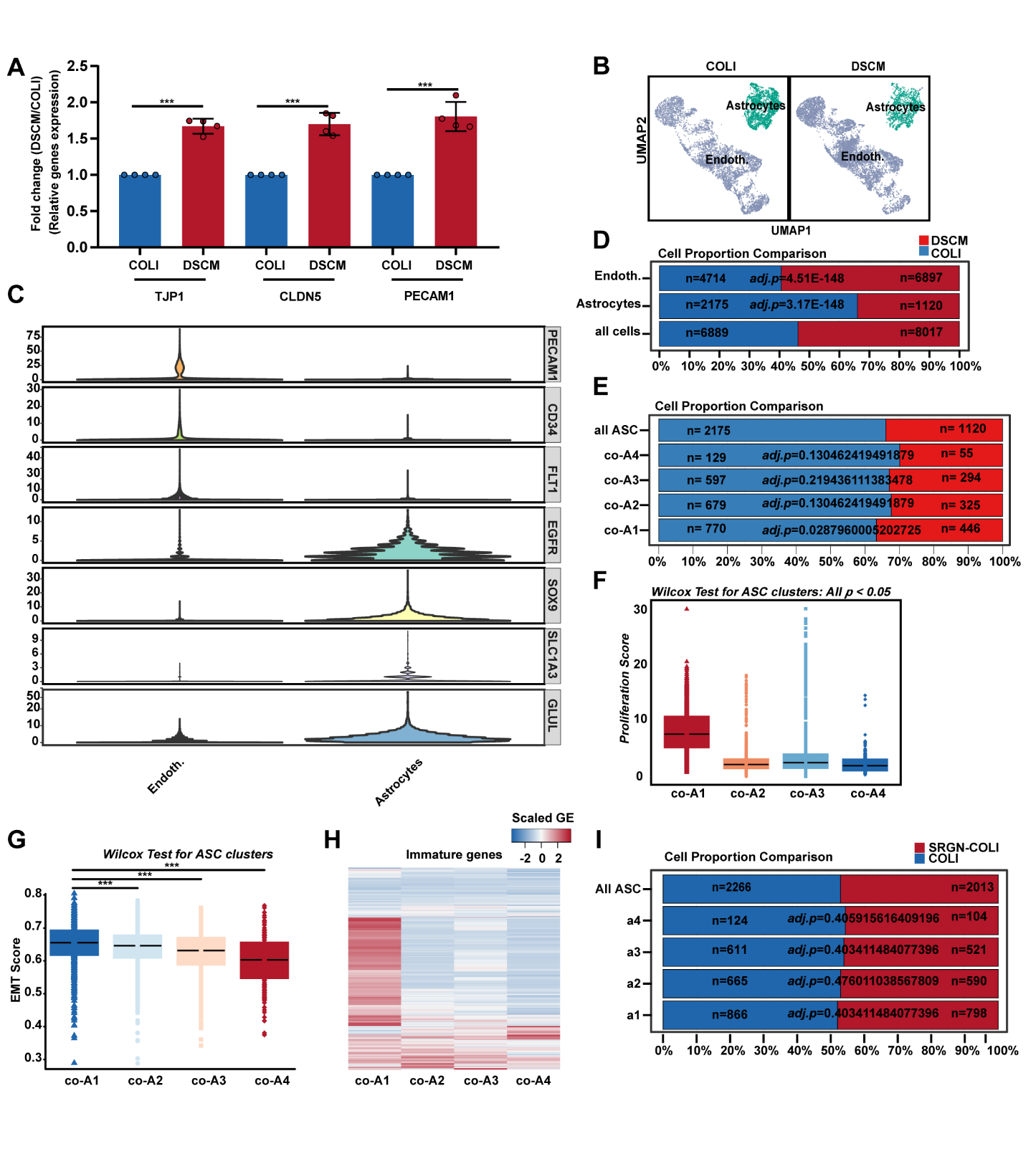


**Fig. S5. Different hydrogel regulates the endothelial and astrocytes subpopulations in the hspASCs-HUVEC co-culture model.** (**A**) qPCR validation of junction-related genes in endothelial cells cultured on DSCM and COLI hydrogel. ***, *p*<0.001 (*t*-test, mean ± SEM). (**B**) UMAP plot integrates the scRNA-seq data from hpsASCs-HUVECs co-culture under COLI and DSCM conditions. (**C**) Violin plot shows the expression of marker genes in the hspASCs-HUVEC co-culture model under DSCM and COLI conditions. (**D**) The bar graph shows the cell proportion comparison of hECs and hspASCs cultured on COLI and DSCM. Cell number (n) and *adj.p* were shown on the graph (hypergeometric tests and Benjamini-Hochberg correction). (**E**) The cell proportion comparison of hspASCs subpopulations. The *adj.p* was calculated based on hypergeometric tests and corrected with Benjamini-Hochberg correction. (**F**), (**G**) Proliferation and EMT score of hspASCs subpopulation in hspASC-HUVEC co-culture model. ***, *p*<0.001. The *p*-value was determined using the Wilcox test. (**H**) Heatmap shows the scaled gene expression of the immature gene set in different hspASCs subtypes cultured on COLI/DSCM. (**I**) Comparison of the proportions of astrocyte subsets in hspASCs-HUVEC co-cultured model under COLI/SRGN-COLI condition. Cell number (n) and *adj. p* are shown on the graph. The *adj p* was calculated based on hypergeometric tests and corrected with Benjamini-Hochberg correction.


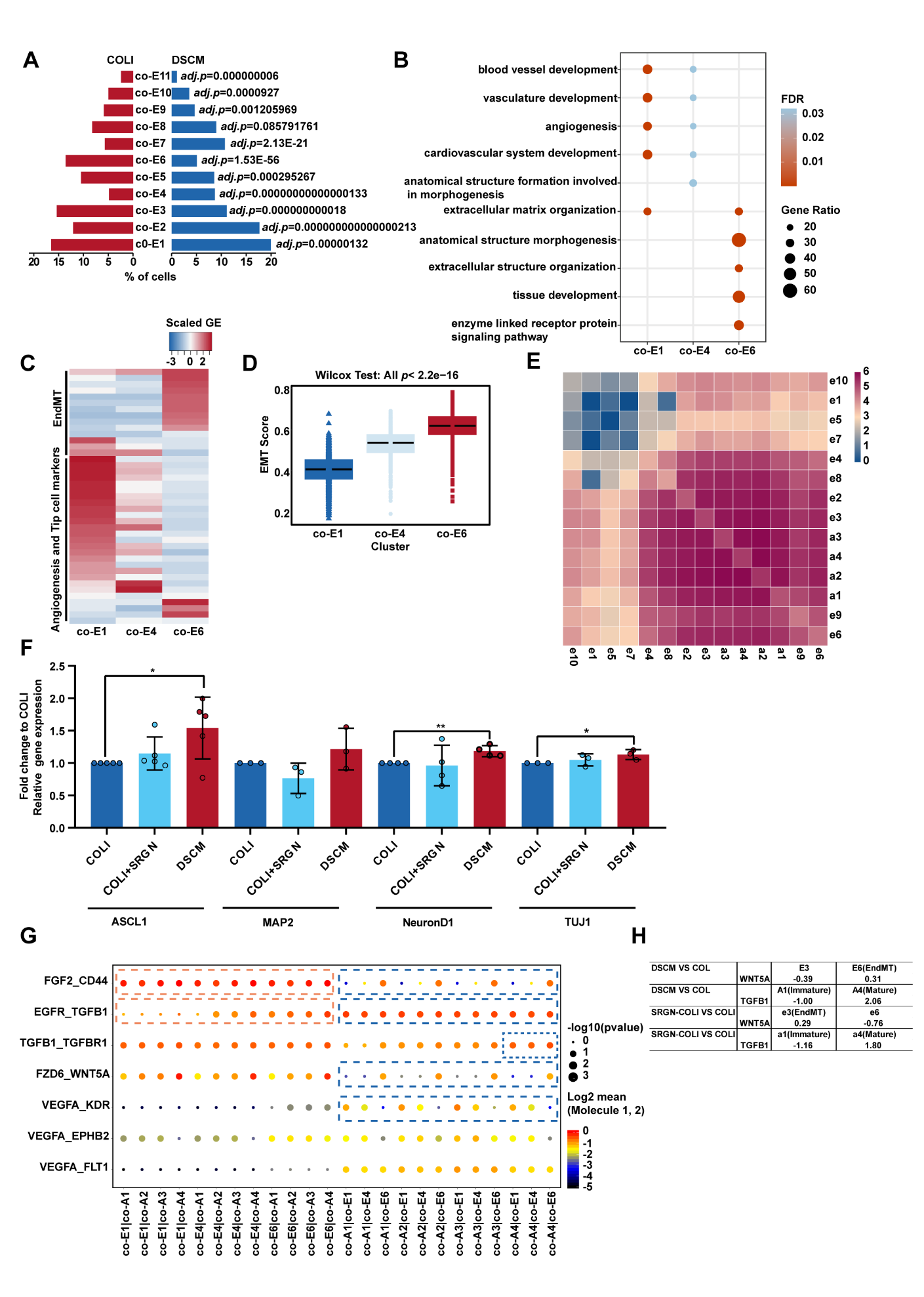


**Fig. S6. Different hydrogel impacts the subtype composition of endothelial in the hspASC-hEC co-culture model. (A)** The bar graph indicates the proportion of endothelial cell subtypes under the COLI and DSCM conditions. The *adj.p* was calculated by hypergeometric tests and corrected with Benjamini-Hochberg correction. (**B**) The dot plot displays the top 5 GO terms of three EC sub-clusters (co-E1, co-E4 and co-E6) based on the top 50 marker genes. (**C**) The heatmap illustrates the gene expression profile of EndMT, Angiogenesis and Tip cell. (**D**) EMT score of endothelial cell subpopulation (co-E1, co-E4 and co-E6) in the hspASCs-HUVEC co-culture system under the COLI and DSCM conditions. ***, *p*<0.001 (Wilcox test). **(E)** The heatmap illustrates the interaction counts between hspASC and hEC subtypes. **(F)** qPCR analysis of the neural differentiation genes in hNSCs under different condition medium. Representative ligand-receptor interactions between HUVEC subtypes (co-E1, co-E4 and co-E6) and hspASC subtypes (co-A1, co-A2, co-A3 and co-A4) were shown in (**G**). (**H**) Scaled gene expression of *WNT5a* and *TGFβ1* in hspASCs and hECs subtypes under different culture conditions.


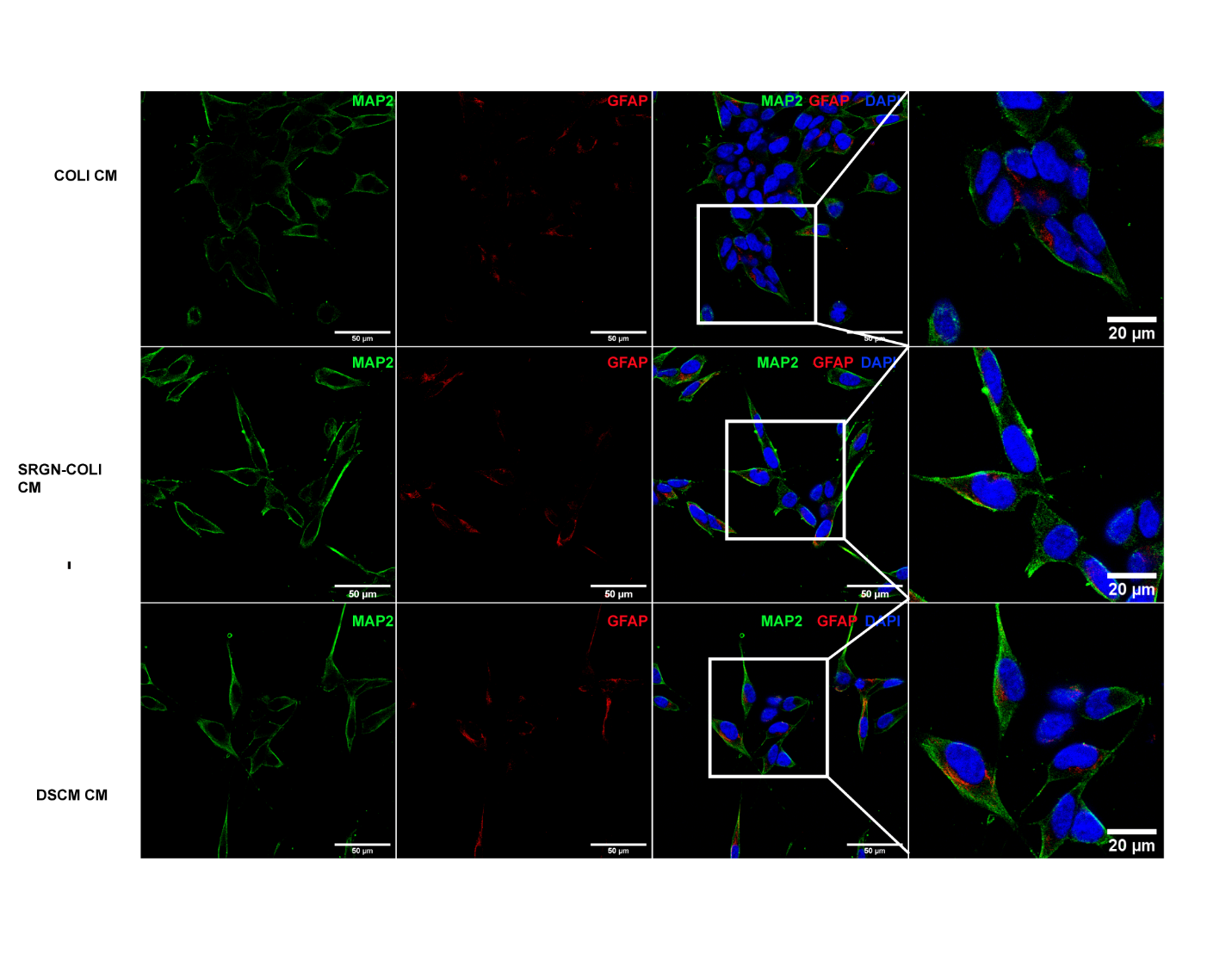


**Fig. S7. Immunofluorescence analysis of MAP2 and GFAP in hNSCs under different condition medium.**

**Table S1** **Primary and secondary antibodies used for western blot**

| Antibodies | Dilution | Source |
| --- | --- | --- |
| GAPDH | 1:10000 | Genetex, GTX100118 |
| EGFR | 1:1000 | Cell Signaling Technology, # 4267S |
| ITGA5 | 1:1000 | Proteintech, 10569-1-AP-100UL |
| GFAP | 1:5000 | Proteintech, 60190-1-IG-100UL |
| CD44 | 1:2000 | Proteintech, 60224-1-IG-100UL |
| Actin | 1:5000 | Proteintech, 66009-1-IG-100UL |
| P-IκBα | 1:1000 | Cell Signaling Technology, #2859S |
| IκBα | 1:1000 | Proteintech, #10268-1-AP |
| TNFR1 | 1:500 | Proteintech, 21574-1-AP |
| CDH2 | 1:1000 | Proteintech, 66219-1-Ig |
| NES | 1:1000 | Proteintech, 19483-1-AP-100UL |
| AKT | 1:1000 | Cell Signaling Technology, #4691S |
| p-AKT | 1:1000 | Cell Signaling Technology, #4060S |
| DCN | 1:1000 | Proteintech, 66847-1-IG-50UL |
| porcine SRGN | 1:1000 | custom Genscript |
| Peroxidase AffiniPure Goat Anti-rabbit IgG (H+L) | 1:10000 | Jackson ImmunoResearch, #111-035-003 |
| Peroxidase AffiniPure Goat Anti-mouse IgG (H+L) | 1:10000 | Jackson ImmunoResearch, #115-035-003 |

**Table S2 qRT-PCR primers**

| Primer | Sequence |
| --- | --- |
| GAPDH-F | GGAGCGAGATCCCTCCAAAAT |
| GAPDH-R | GGCTGTTGTCATACTTCTCATGG |
| NES-F | CTGGAGCAGGAGAAACAGG |
| NES-R | TGGGAGCAAAGATCCAAGAC |
| GFAP-F | CTGCGGCTCGATCAACTCA |
| GFAP-R | TCCAGCGACTCAATCTTCCTC |
| CDH2-F | AGCCAACCTTAACTGAGGAGT |
| CDH2-R | GGCAAGTTGATTGGAGGGATG |
| Ki67-F | GAAAGAGTGGCAACCTGCCTTC |
| Ki67-R | GCACCAAGTTTTACTACATCTGCC |
| CD44-F | TCCATCAAAGGCATTGGGCAG |
| CD44-R | AACCTGCCGCTTTGCAGGTGT |
| CyclinD1-F | TATTGCGCTGCTACCGTTGA |
| CyclinD1-R | CCAATAGCAGCAAACAATGTGAAA |
| EGFR-F | AGGCACGAGTAACAAGCTCAC |
| EGFR-R | ATGAGGACATAACCAGCCACC |
| TPJ1-F | CAACATACAGTGACGCTTCACA |
| TPJ1-R | CACTATTGACGTTTCCCCACTC |
| CLDN5-F | CTCTGCTGGTTCGCCAACAT |
| CLDN5-R | CAGCTCGTACTTCTGCGACA |
| MMP2-F | CCCACTGCGGTTTTCTCGAAT |
| MMP2-R | CAAAGGGGTATCCATCGCCAT |
| IL-6-F | TACCCCCAGGAGAAGATTCC |
| IL-6-R | GCCATCTTTGGAAGGTTCAG |
| TGFβ1-F | CGACTCGCCAGAGTGGTTAT |
| TGFβ1-R | TAGTGAACCCGTTGATGTCCA |
| VIM-F | CGAAAACACCCTGCAATCTT |
| VIM-R | AAGGTCAAGACGTGCCAGA |
| FN1-F | AACAAACACTAATGTTAATTGCCCA |
| FN1-R | TCGGGAATCTTCTCTGTCAGC |
| IL-1b-F | GCTGAGGAAGATGCTGGTTC |
| IL-1b-R | TCCATATCCTGTCCCTGGAG |
| AKT1-F  AKT1-R  HK2-F  HK2-R  PFKP-F  PFKP-R  PKM-F  PKM-R  LDHA-F  LDHA-R | AGCGACGTGGCTATTGTGAAG  GCCATCATTCTTGAGGAGGAAGT  GAGCCACCACTCACCCTACT  CCAGGCATTCGGCAATGTG  GACCTTCGTTCTGGAGGTGAT CACGGTTCTCCGAGAGTTTG  ATAACGCCTACATGGAAAAGTGT  TAAGCCCATCATCCACGTAGA  ATGGCAACTCTAAAGGATCAGC  CCAACCCCAACAACTGTAATCT |

**Table S3 Primary and secondary antibodies used for immunofluorescence staining**

| Antibodies | Species | Dilution | Source |
| --- | --- | --- | --- |
| CDH5 | Mouse | 1:100 | Proteintech, 66804-1-Ig |
| Glial fibrillary acidic protein (GFAP) | Rabbit | 1:500 | Dako, z0034 |
| NESTIN | Rabbit | 1:100 | Proteintech, 19483-1-AP-100UL |
| SOX2 | Mouse | 1:100 | R&D, MAB2018 |
| CD31 | Sheep | 1:100 | R&D Systems, AF806 |
| Alexa Fluor 488 goat anti-mouse antibody | Goat | 1:1000 | Invitrogen, A11001 |
| Alexa Fluor 488 goat anti-rabbit antibody | Goat | 1:1000 | Invitrogen, A11008 |
| Alexa Fluor 568 goat anti-rabbit antibody | Goat | 1:1000 | Invitrogen, A11036 |
| Alexa Fluor 488 donkey anti-sheep antibody | Donkey | 1:1000 | Invitrogen, A11015 |
